# Association of Bariatric Surgery With Cardiovascular Outcomes in Adults With Severe Obesity and Nonalcoholic Fatty Liver Disease

**DOI:** 10.1001/jamanetworkopen.2022.35003

**Published:** 2022-10-07

**Authors:** Mohamed I. Elsaid, You Li, John F. P. Bridges, Guy Brock, Carlos D. Minacapelli, Vinod K. Rustgi

**Affiliations:** 1 Department of Biomedical Informatics, College of Medicine, The Ohio State University, Columbus; 2 Secondary Data Core, Center for Biostatistics, College of Medicine, The Ohio State University, Columbus; 3 Division of Gastroenterology and Hepatology, Department of Medicine, Rutgers Robert Wood Johnson Medical School, New Brunswick, New Jersey; 4 Department of Surgery, College of Medicine, The Ohio State University, Columbus; 5 Center for Liver Diseases and Masses, Rutgers Robert Wood Johnson Medical School, New Brunswick, New Jersey

## Abstract

**Importance:**

There are no approved treatments for nonalcoholic fatty liver disease (NAFLD) despite its association with obesity and increased risk of cardiovascular disease (CVD).

**Objective:**

To examine the association between bariatric surgery and CVD risk in individuals with severe obesity and NAFLD.

**Design, Setting, and Participants:**

This large, population-based retrospective cohort study obtained data from the MarketScan Commercial Claims and Encounters database from January 1, 2007, to December 31, 2017. Participants included insured adults aged 18 to 64 years with NAFLD and severe obesity (body mass index ≥40) without a history of bariatric surgery or CVD before NAFLD diagnosis. Baseline characteristics were balanced between individuals who underwent surgery (surgical group) and those who did not (nonsurgical group) using inverse probability of treatment weighting. Data were analyzed from March 2020 to April 2021.

**Exposures:**

Bariatric surgery (Roux-en-Y gastric bypass, sleeve gastrectomy, and other bariatric procedures) vs nonsurgical care.

**Main Outcomes and Measures:**

The main outcome was the incidence of cardiovascular events (primary or secondary composite CVD outcomes). The primary composite outcome included myocardial infarction, heart failure, or ischemic stroke, and the secondary composite outcome included secondary ischemic heart events, transient ischemic attack, secondary cerebrovascular events, arterial embolism and thrombosis, or atherosclerosis. Cox proportional hazards regression models with inverse probability treatment weighting were used to examine the associations between bariatric surgery, modeled as time varying, and all outcomes.

**Results:**

The study included 86 964 adults (mean [SD] age, 44.3 [10.9] years; 59 773 women [68.7%]). Of these individuals, 30 300 (34.8%) underwent bariatric surgery and 56 664 (65.2%) received nonsurgical care. All baseline covariates were balanced after applying inverse probability treatment weighting. In the surgical group, 1568 individuals experienced incident cardiovascular events compared with 7215 individuals in the nonsurgical group (incidence rate difference, 4.8 [95% CI, 4.5-5.0] per 100 person-years). At the end of the study, bariatric surgery was associated with a 49% lower risk of CVD (adjusted hazard ratio [aHR], 0.51; 95% CI, 0.48-0.54) compared with nonsurgical care. The risk of primary composite CVD outcomes was reduced by 47% (aHR, 0.53 [95% CI, 0.48-0.59), and the risk of secondary composite CVD outcomes decreased by 50% (aHR, 0.50; 95% CI, 0.46-0.53) in individuals with vs without surgery.

**Conclusions and Relevance:**

Results of this study suggest that, compared with nonsurgical care, bariatric surgery was associated with significant reduction in CVD risk in individuals with severe obesity and NAFLD.

## Key Points

**Question:**

Is bariatric surgery a factor in reducing the risk of cardiovascular outcomes in adults with severe obesity and nonalcoholic fatty liver disease (NAFLD)?

**Findings:**

In this large cohort study of 86 964 individuals with NAFLD and severe obesity, compared with nonsurgical care, bariatric surgery was associated with a 49% decrease in the risk of cardiovascular disease, a 47% decrease in the risk of primary composite cardiovascular events, and a 50% lower risk of secondary composite cardiovascular events.

**Meaning:**

Findings of this study suggest that individuals with severe obesity and NAFLD who underwent bariatric surgery have a lower cardiovascular disease risk than those who received nonsurgical care.

## Teaser

This cohort study compares the risk of experiencing cardiovascular events after bariatric surgery vs nonsurgical treatment in adults across the full spectrum of fatty liver disease.

## Introduction

Nonalcoholic fatty liver disease (NAFLD) is the most common chronic liver disease in the US, affecting more than 30% of adults.^1^ It is characterized by hepatic steatosis in the absence of substantial alcohol consumption, long-term use of steatogenic drugs, or genetic disorders.^2^ The disease encompasses the full spectrum of fatty liver disease, including nonalcoholic fatty liver, nonalcoholic steatohepatitis (NASH), advanced fibrosis, and cirrhosis.^2,3^ Although individuals with NAFLD are typically asymptomatic, NASH has been associated with increased risk of advanced fibrosis, NASH-related cirrhosis, and hepatocellular carcinoma.^4,5,6^

Nonalcoholic fatty liver disease is the hepatic manifestation of metabolic syndrome because it is closely linked to obesity-induced insulin resistance, dyslipidemia, and hypertension.^1,7,8,9,10^ The prevalence of NAFLD increases with body mass index (BMI) and is highest among individuals with severe obesity (BMI ≥40 [calculated as weight in kilograms divided by height in meters squared]), of whom 85% had NAFLD and 40% had NASH.^11^ Although NAFLD and cardiovascular disease (CVD) share common risk factors,^12,13,14^ NAFLD is an important risk factor for CVD morbidity and mortality independent of the traditional factors associated with CVD.^15,16,17,18,19,20,21^

There are no approved pharmacological treatments for NAFLD despite its association with increased risk of CVD-related morbidity and mortality.^22^ Lifestyle modifications, such as weight loss, healthier diet, and regular exercise, are associated with improved hepatic steatosis and cardiometabolic indices in NAFLD.^23,24,25^ However, the benefits of these modifications have proven difficult to sustain.^23,24,26^ Although bariatric surgery has been associated with long-term improvements in NAFLD histological features^27,28,29,30^ and reductions in CVD risk in individuals with obesity,^31,32,33,34^ the association between bariatric surgery and CVD risk has not been thoroughly investigated in the full NAFLD spectrum. To our knowledge, only 1 study to date has investigated the association.^35^ However, that study was limited to a small sample of individuals with NASH in whom a modest number of CVD events were observed.

To address this knowledge gap, we conducted a large, population-based retrospective cohort study to examine the association between bariatric surgery and CVD risk in individuals with severe obesity and NAFLD. We hypothesized that bariatric surgery would be associated with lower CVD risk. Furthermore, we hypothesized that individuals with NAFLD who underwent bariatric surgery would have lower risks of primary and secondary CVD outcomes than those who received nonsurgical care. We believe the findings of this study help to examine the effectiveness of bariatric surgery in reducing the elevated CVD risk in individuals with severe obesity and NAFLD for whom lifestyle modifications were not sustainable.

## Methods

In this cohort study, we obtained data on adults aged 18 to 64 years with NAFLD and severe obesity using the MarketScan Commercial Claims and Encounters database (IBM Watson Health) from January 1, 2007, to December 31, 2017. MarketScan is a nationwide database with deidentified, individual-level claims records from outpatient, inpatient, and prescription drug services for more than 230 million privately insured enrollees and dependents in the US.^36^ The MarketScan database is widely used in epidemiological, clinical, and outcomes research.^37,38,39,40,41^ Rutgers Robert Wood Johnson Medical School Institutional Review Board approved the study protocol and waived the informed consent requirement because the study used a commercial database with deidentified records. We followed the Strengthening the Reporting of Observational Studies in Epidemiology (STROBE) reporting guideline.

### Study Cohort

The sample included insured adults with at least 1 inpatient or outpatient NAFLD diagnosis. We used a validated diagnostic algorithm with an 85% positive predictive value to identify individuals with NAFLD (eTable 1 in the Supplement).^42^ We limited the study cohort to adults with a minimum of 12 months of continuous insurance enrollment before the first NAFLD diagnosis (index date). We excluded individuals with any records for other liver diseases, excessive alcohol use, bariatric surgery, or any of the study’s CVD outcomes before the index date. To reflect the clinical guidelines implemented during the study period and the nature of the administrative data used in the analysis, we limited the sample to adults with NAFLD and severe obesity (BMI ≥40), resulting in the base cohort (eMethods 1 in the Supplement).

We queried the records of all individuals in the base cohort to identify who underwent bariatric surgery. We evaluated only primary procedures performed after the index date to avoid misclassifications. All bariatric surgeries, including Roux-en-Y gastric bypass (RYGB), sleeve gastrectomy, and other bariatric procedures were defined using the procedure codes suggested by the American Society for Metabolic and Bariatric Surgery (eTable 2 in the Supplement).^43^ Starting with the base cohort, we assigned those with at least 1 primary record for bariatric surgery after the index date to the surgical group.

### Outcomes

The main outcome was the incidence of cardiovascular events (CVEs), defined as the first occurrence of either primary or secondary composite CVD outcomes. The primary composite outcome included myocardial infarction (MI), heart failure, or ischemic stroke. The secondary composite cardiovascular outcome included either (1) secondary ischemic heart events, including angina pectoris, complications of MI, acute coronary thrombosis, Dressler syndrome, or chronic ischemic heart diseases; (2) transient ischemic attack; (3) secondary cerebrovascular events, including occlusion and stenosis of precerebral or cerebral arteries not resulting in ischemic stroke, cerebral atherosclerosis, acute cerebrovascular insufficiency, or cerebral ischemia; (4) arterial embolism and thrombosis; or (5) atherosclerosis (eTable 2 in the Supplement).

The follow-up period was measured from the index date to the event date for participants with CVD outcomes. For participants without CVD outcomes, the follow-up period was from the index date to the end of study enrollment or end of the study, whichever occurred first. For participants with both primary and secondary outcomes, if an individual had a secondary event before a primary event diagnosis, the individual was assumed to have had a primary event when assessing the risk of primary outcomes. In evaluating the risk of the secondary outcomes, if an individual had a primary event before the secondary event, the individual was assumed to have had a primary outcome and was censored at the primary event date. We conducted sensitivity analyses to assess those assumptions.

### Statistical Analysis

Bariatric surgery status (surgical vs nonsurgical) was allowed to be time varying to account for the implication of the wait time between the index date and operation date (eFigure 1 in the Supplement). We used standardized inverse probability of treatment weighting (IPTW) to account for the implications of confounding by indication, which results from the differences in baseline characteristics and medical histories that affect the appropriateness of the surgery.^44^ The IPTW for each participant was estimated using a multivariable logistic regression model with surgery status as the outcome and all of the included variables (Table 1) as covariates. The study was restricted to participants with overlapping propensity scores, and we compared participants’ baseline characteristics and medical histories by bariatric surgery status using standardized differences with IPTW.

**Table 1.** Baseline Characteristics of the Study Sample by Bariatric Surgery Status

Characteristic	Individuals, No. (%) (N = 86 964)		Standardized difference	
	Surgical group (n = 30 300)	Nonsurgical group (n = 56 664)	Unweighted^a^	Weighted^b^
Demographic^c^				
Age, y				
Mean (SD)	43.3 (10.3)	44.9 (11.2)	0.152	0.052
Median (IQR)	44.0 (37.0-51.0)	46.0 (37.0-54.0)		
Age group, y				
18-34	6413 (21.2)	11 142 (19.7)	0.037	0.004
35-44	9760 (32.2)	14 837 (26.2)	0.133	0.002
45-54	9397 (31.0)	17 743 (31.3)	0.007	0.004
≥55	4730 (15.6)	12 942 (22.8)	0.184	0.002
Sex				
Male	7305 (24.1)	19 886 (35.1)	0.242	0.006
Female	22 995 (75.9)	36 778 (64.9)	0.242	0.006
Region of residence				
Northeast	6136 (20.3)	9753 (17.2)	0.078	0.004
North Central	5349 (17.7)	10 954 (19.3)	0.043	0.007
South	12 798 (42.2)	25 176 (44.4)	0.044	0.001
West	5526 (18.2)	9786 (17.3)	0.025	0.001
Unknown	491 (1.6)	995 (1.8)	0.011	0.002
Type of health insurance				
PPO	19 324 (63.8)	34 371 (60.7)	0.064	0.001
HMO	3203 (10.6)	6889 (12.2)	0.050	0.007
Comprehensive	570 (1.9)	1063 (1.9)	0.001	0.001
POS with capitation	2765 (9.1)	4382 (7.7)	0.050	0.005
Other	4438 (14.7)	9959 (17.6)	0.078	0.009
Year of NAFLD diagnosis				
2008	1526 (5.0)	1039 (1.8)	0.177	0.001
2009	3462 (11.4)	3242 (5.7)	0.205	0.002
2010	3569 (11.8)	3598 (6.4)	0.190	0.005
2011	3751 (12.4)	4397 (7.8)	0.154	0.005
2012	4080 (13.5)	6234 (11.0)	0.075	0.006
2013	3390 (11.2)	5232 (9.2)	0.065	0.002
2014	3464 (11.4)	7241 (12.8)	0.041	0.002
2015	2632 (8.7)	6705 (11.8)	0.104	0.001
2016	2494 (8.2)	9015 (15.9)	0.237	0.004
2017	1932 (6.4)	9961 (17.6)	0.350	0.015
History of smoking	1862 (6.2)	5342 (9.4)	0.123	0.001
Medical history^d^				
Asthma	4206 (13.9)	7586 (13.4)	0.014	0.010
Obstructive sleep apnea	10 455 (34.5)	13 570 (24.0)	0.234	0.008
Obesity hypoventilation syndrome	108 (0.36)	232 (0.41)	0.009	0.001
Severe urinary incontinence	1252 (4.1)	1670 (3.0)	0.064	0.003
Chronic venous insufficiency	527 (1.7)	1115 (2.0)	0.017	0.002
Osteoarthritis	4991 (16.5)	9156 (16.2)	0.009	0.009
Diabetes	9882 (32.6)	19 013 (33.6)	0.020	0.004
Hypertension	17 029 (56.2)	32 549 (57.4)	0.025	0.006
Dyslipidemia	13 668 (45.1)	25 904 (45.7)	0.012	0.008
CKD	350 (1.2)	1156 (2.0)	0.071	0.003
Cancer	2802 (9.3)	7064 (12.5)	0.104	0.001
Cirrhosis	832 (2.8)	1361 (2.4)	0.022	0.018

Abbreviations: CKD, chronic kidney disease; HMO, health maintenance organization; NAFLD, nonalcoholic fatty liver disease; POS, point of service; PPO, preferred provider organization.

^a^ Absolute difference in means or proportions divided by pooled SD. Imbalance between the surgical and nonsurgical groups was defined as an absolute value greater than 0.10; smaller values indicated better balance.

^b^ Inverse probability of treatment weighted standardized differences. All demographic characteristics and medical histories were used to estimate the weights.

^c^ Obtained on the NAFLD index date.

^d^ Obtained from the 12 months before the NAFLD index date.

Cumulative incidences were estimated at 24, 48, 72, and 96 months of follow-up using the Simon and Makuch method to account for bariatric surgery status that was modeled as time varying.^45^ We performed Mantel and Byar tests for survival comparisons of time-varying data to compare participants’ survival probabilities according to surgery status.^46^ We used IPTW-adjusted Cox proportional hazards regression models with robust variance to examine the associations between bariatric surgery status and the study outcomes. We also tested the interactions between bariatric surgery status and demographic characteristics and medical histories. The MarketScan database did not include race and ethnicity data.

Cause-specific proportional hazards regression models were used to assess the associations between surgery status and the 8 individual components of the primary and secondary CVD outcomes. All cause-specific models accounted for competing risks by censoring follow-up time at the first date of any cardiovascular diagnosis or in-hospital mortality regardless of the type of event.

A level of *P* = .05 for 2-sided tests was considered to be statistically significant. All analyses were performed using SAS, version 9.4 (SAS Institute Inc). Data were analyzed from March 2020 to April 2021.

We conducted several sensitivity analyses to assess the robustness of the main findings (eMethods 2 in the Supplement). First, we included the secondary composite CVD outcome as a time-varying covariate in the primary outcome analysis to account for the occurrence of the secondary outcome before primary events. Second, we redefined the incidence of all CVD outcomes as the presence of at least 2 separate inpatient or outpatient claims made 90 days or more after NAFLD diagnosis. Third, we extended the sample to all individuals in the MarketScan database with NAFLD and a BMI of 35 or higher. Fourth, we limited bariatric surgeries to RYGB and sleeve gastrectomy. Fifth, we used inverse probability of censoring weighting to examine the outcomes of potential selection bias associated with informative censoring. Sixth, we calculated E-values and bias factors to assess the robustness of the main findings against potential unmeasured confounders.^47^

## Results

The study included 86 964 adults (mean [SD] age, 44.3 [10.9] years; 59 773 women [68.7%] and 27 191 men [31.3%]). Of these individuals, 30 300 (34.8%) underwent bariatric surgery (surgical group) and 56 664 (65.2%) received nonsurgical care (nonsurgical group) (eFigure 2 in the Supplement). Those who received surgery had a mean 7.2 months wait between the index date and surgery date, and 28 608 of these individuals did not have an outcome before receiving surgery and were categorized in the surgical group in all analyses. The study sample included 11 371 RYGBs, 10 404 sleeve gastrectomies, and 8525 other bariatric surgeries (eFigure 3 in the Supplement). The mean (SD) follow-up time for all participants was 21.1 (20.7) months, with 29.2 (24.6) months for those in the surgical group and 16.8 (16.8) months for those in the nonsurgical group.

Compared with those in the nonsurgical group, individuals in the surgical group were younger (43.3 vs 44.9 years; *P* < .001), more likely to be women (75.9% vs 64.9%; *P* < .001), and less likely to have a history of smoking (6.2% vs 9.4%; *P* < .001). All estimated IPTW-adjusted standardized differences were lower than the 0.1 thresholds, indicating negligible baseline differences between the surgical group and nonsurgical group (Table 1). In the IPTW-adjusted sample, the associations between bariatric surgery status and baseline characteristics and medical histories were statically insignificant except for cirrhosis, which remained more prevalent among those in the surgical group (eTable 3 in the Supplement).

### CVD Risk After Bariatric Surgery

**Figure 1.**
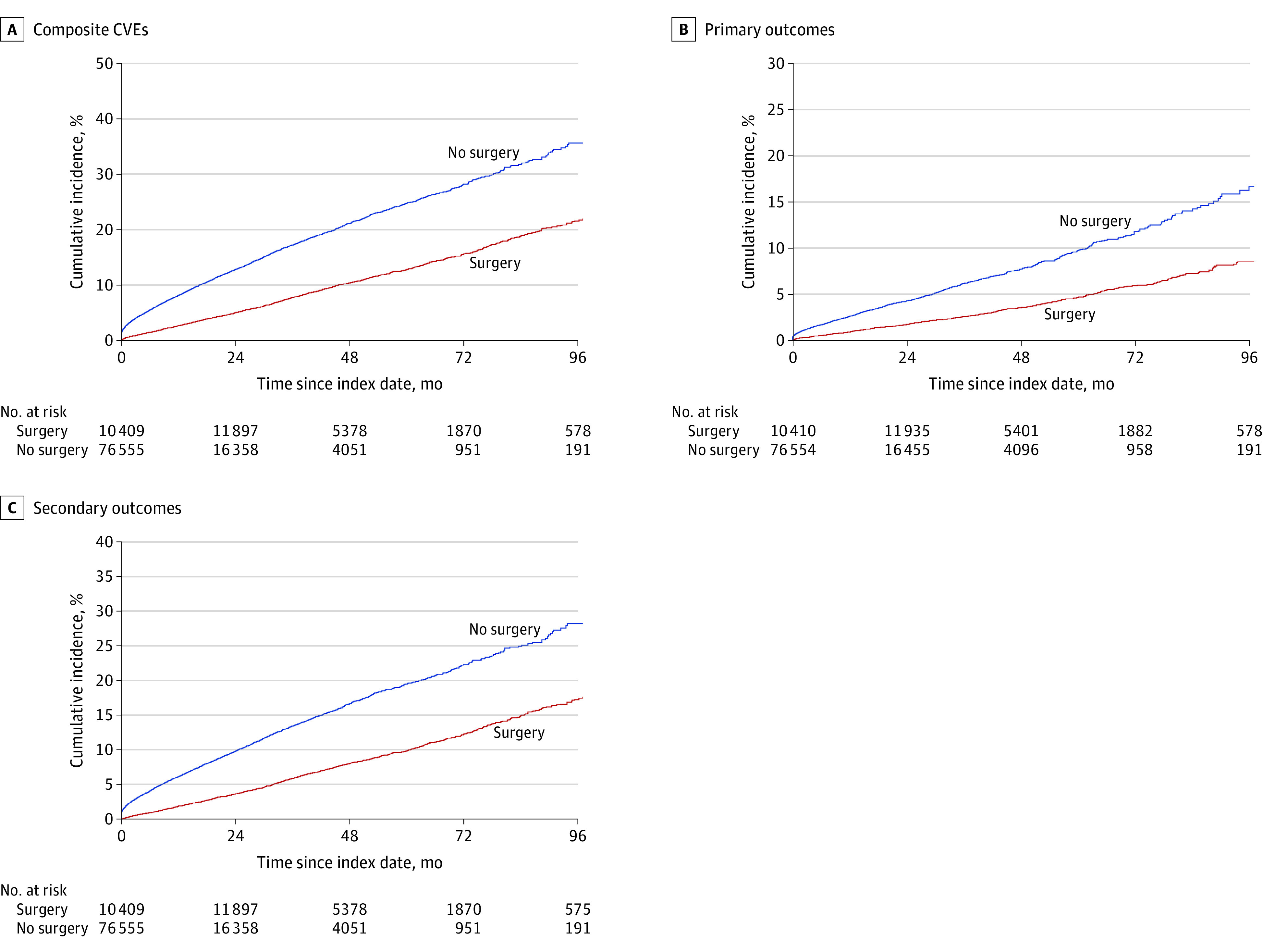
Cumulative Incidence of Composite Cardiovascular Events (CVEs), Primary Composite Cardiovascular Disease (CVD) Outcome, and Secondary Composite CVD Outcome by Bariatric Surgery Status in Adults With Nonalcoholic Fatty Liver Disease and Severe Obesity Individuals who received nonsurgical care (n = 58 356) and individuals with surgical care (n = 28 608) experienced 7215 and 1568 incidences of CVEs (A), 2401 and 549 incidences of primary composite CVD outcome (B), and 5424 and 1191 incidences of secondary composite CVD outcome (C), respectively. Bariatric surgery status was modeled as a time-varying variable. Survival estimates were obtained using the Simon-Makuch method. The Mantel and Byar test for survival comparisons of data with a time-varying covariate had *P* < .001 for differences between surgical and nonsurgical groups in all panels.

Bariatric surgery was associated with a significantly lower risk of incident CVEs (Figure 1A). At the 96-month follow-up, the surgical group had 1568 incident CVEs over 57 061.4 person-years, whereas the nonsurgical group had 7215 CVD cases over 96 150.1 person-years (incidence rate difference, 4.8 [95% CI, 4.5-5.0] per 100 person-years) (Table 2). In the surgical group, the cumulative incidences of CVEs were 5.0% at 24 months, 10.4% at 48 months, 15.6% at 72 months, and 21.6% at 96 months. In the nonsurgical group, the cumulative incidences of CVEs were 12.8% at 24 months, 21.1% at 48 months, 28.2% at 72 months, and 35.6% at 96 months (eTable 4 in the Supplement).

**Table 2.** Associations Between Bariatric Surgery Status and Risk of Composite Cardiovascular Events (CVEs) in Adults With Nonalcoholic Fatty Liver Disease and Severe Obesity^a^

Variable	No. of participants	No. of events	Person-years	Incidence rate, 100 person-years (95% CI)	Absolute rate difference, 100 person-years (95% CI)	Adjusted HR (95% CI)^b^**^,^**^**c**^
Composite CVE^d^						
Without bariatric surgery	58 356	7215	96 150.1	7.5 (7.3-7.7)	1 [Reference]	1 [Reference]
With bariatric surgery	28 608	1568	57 061.4	2.8 (2.6-2.9)	4.8 (4.5-5.0)	0.51 (0.48-0.54)
Age group						
18-34 y						
Without bariatric surgery	11 301	507	18 690.8	2.7 (2.5-3.0)	1 [Reference]	1 [Reference]
With bariatric surgery	6254	135	11 411.4	1.2 (1.0-1.4)	1.5 (1.2-1.8)	0.54 (0.44-0.67)
35-44 y						
Without bariatric surgery	15 263	1416	27 098.7	5.2 (5.0-5.5)	1 [Reference]	1 [Reference]
With bariatric surgery	9334	396	18 795.0	2.1 (1.9-2.3)	3.1 (2.8-3.5)	0.53 (0.47-0.60)
45-54 y						
Without bariatric surgery	18 445	2786	31 739.0	8.8 (8.5-9.1)	1 [Reference]	1 [Reference]
With bariatric surgery	8695	607	18 342.6	3.3 (3.1-3.6)	5.5 (5.1-5.9)	0.47 (0.43-0.52)
≥55 y						
Without bariatric surgery	13 347	2506	18 621.6	13.5 (12.9-14.0)	1 [Reference]	1 [Reference]
With bariatric surgery	4325	430	8512.4	5.1 (4.6-5.6)	8.4 (7.7-9.1)	0.50 (0.45-0.55)
Sex						
Male						
Without bariatric surgery	20 378	2635	31 833.9	8.3 (8.0-8.6)	1 [Reference]	1 [Reference]
With bariatric surgery	6813	408	13 472.0	3.0 (2.7-3.3)	5.2 (4.8-5.7)	0.55 (0.50-0.61)
Female						
Without bariatric surgery	37 978	4580	64 316.2	7.1 (6.9-7.3)	1 [Reference]	1 [Reference]
With bariatric surgery	21 795	1160	43 589.3	2.7 (2.5-2.8)	4.5 (4.2-4.7)	0.49 (0.45-0.53)
Asthma						
Without bariatric surgery	7825	1133	12 106.3	9.4 (8.8-9.9)	1 [Reference]	1 [Reference]
With bariatric surgery	3967	248	7399.6	3.4 (3.0-3.8)	6.0 (5.3-6.7)	0.48 (0.41-0.55)
Obstructive sleep apnea						
Without bariatric surgery	14 017	2019	20 855.2	9.7 (9.3-10.1)	1 [Reference]	1 [Reference]
With bariatric surgery	10 008	577	19 090.5	3.0 (2.8-3.3)	6.6 (6.2-7.1)	0.43 (0.39-0.48)
Obesity hypoventilation syndrome						
Without bariatric surgery	234	41	222.1	18.5 (13.3-25.1)	1 [Reference]	1 [Reference]
With bariatric surgery	106	4	123.9	3.2 (0.9-8.3)	15.2 (8.8-21.7)	0.24 (0.07-0.62)
Severe urinary incontinence						
Without bariatric surgery	1733	269	2584.1	10.4 (9.2-11.7)	1 [Reference]	1 [Reference]
With bariatric surgery	1189	71	2181.5	3.3 (2.5-4.1)	7.2 (5.7-8.6)	0.43 (0.32-0.58)
Chronic venous insufficiency						
Without bariatric surgery	1146	236	1537.4	15.4 (13.5-17.4)	1 [Reference]	1 [Reference]
With bariatric surgery	496	41	981.9	4.2 (3.0-5.7)	11.2 (8.8-13.5)	0.40 (0.28-0.56)
Osteoarthritis						
Without bariatric surgery	9487	1598	13 814.7	11.6 (11.0-12.2)	1 [Reference]	1 [Reference]
With bariatric surgery	4660	326	8710.4	3.7 (3.4-4.2)	7.8 (7.1-8.5)	0.41 (0.36-0.47)
Type 2 diabetes						
Without bariatric surgery	19 742	3306	30 751.3	10.8 (10.4-11.1)	1 [Reference]	1 [Reference]
With bariatric surgery	9153	691	18 257.7	3.8 (3.5-4.1)	7.0 (6.5-7.4)	0.47 (0.43-0.52)
Hypertension						
Without bariatric surgery	33 607	5041	52 014.4	9.7 (9.4-10.0)	1 [Reference]	1 [Reference]
With bariatric surgery	15 971	1045	31 480.5	3.3 (3.1-3.5)	6.4 (6.0-6.7)	0.48 (0.45-0.51)
Dyslipidemia						
Without bariatric surgery	26 756	3959	42 686.8	9.3 (9.0-10.0)	1 [Reference]	1 [Reference]
With bariatric surgery	12 816	837	25 097.9	3.3 (3.1-3.6)	5.9 (5.6-6.3)	0.50 (0.46-0.54)
CKD						
Without bariatric surgery	1188	276	1574.7	17.5 (15.5-19.7)	1 [Reference]	1 [Reference]
With bariatric surgery	318	44	514.4	8.6 (6.2-11.5)	9.0 (5.7-12.2)	0.56 (0.41-0.75)
Cancer						
Without bariatric surgery	7324	1388	13 283.3	10.5 (9.9-11.0)	1 [Reference]	1 [Reference]
With bariatric surgery	2542	282	5896.3	4.8 (4.2-5.4)	5.7 (4.9-6.5)	0.64 (0.56-0.73)
Cirrhosis						
Without bariatric surgery	1361	310	2048.2	15.1 (13.5-16.9)	1 [Reference]	1 [Reference]
With bariatric surgery	832	80	1685.9	4.8 (3.8-5.9)	10.4 (8.4-12.4)	0.40 (0.31-0.51)

Abbreviations: CKD, chronic kidney disease; HR, hazard ratio.

^a^ Bariatric surgery status was modeled as a time-varying covariate.

^b^ Using inverse probability of treatment weighting and adjusted for age, type of health insurance, region of residence, year of nonalcoholic fatty liver disease diagnosis, sex, smoking status, asthma, obstructive sleep apnea, obesity hypoventilation syndrome, severe urinary incontinence, chronic venous insufficiency, osteoarthritis, diabetes, hypertension, dyslipidemia, CKD, and cancer.

^c^ No significant interaction (*P* > .05) between any of the listed variables and surgery status.

^d^ Myocardial infarction, heart failure, ischemic stroke, secondary ischemic heart events (angina pectoris, complications of myocardial infarction, acute coronary thrombosis, Dressler syndrome, or chronic ischemic heart diseases), transient ischemic attack, secondary cerebrovascular events (occlusion and stenosis of precerebral or cerebral arteries not resulting in ischemic stroke, cerebral atherosclerosis, acute cerebrovascular insufficiency, or cerebral ischemia), arterial embolism and thrombosis, or atherosclerosis.

The IPTW-adjusted hazard of CVEs was significantly lower (by 49%) in individuals with NAFLD who underwent surgery than in those treated nonsurgically (adjusted hazard ratio [aHR], 0.51; 95% CI, 0.48-0.54). None of the interactions between surgery status and demographic characteristics and medical histories were statistically significant.

### Primary CVD Outcomes After Bariatric Surgery

We observed 2950 primary CVD events, of which 784 followed a secondary CVD event. The risk of the primary incident event was significantly lower in the surgical than in the nonsurgical group (Figure 1B). The incidence rate of the primary outcomes was also lower for individuals with vs without surgery status (absolute rate difference, 15.3 [95% CI, 14.0-16.6] per 1000 person-years) (Table 3). At the 96-month follow-up, bariatric surgery was associated with a 47% lower cumulative incidence of primary events (9.7% for surgical group vs 18.3% for nonsurgical group; aHR, 0.53 [95% CI, 0.48-0.59]) (eTable 4 in the Supplement; Table 3). The hazard of primary CVD outcomes remained significantly lower in individuals in the surgical group after adjusting for secondary events occurring before the primary outcomes (aHR, 0.61; 95% CI, 0.55-0.67).

**Table 3.** Associations Between Bariatric Surgery Status and Risk of CVD Outcomes in Adults With Nonalcoholic Fatty Liver Disease and Severe Obesity^a^

Outcome	No. of participants	No. of events	Person-years	Incidence rate, 1000 person-years (95% CI)	Absolute rate difference, 1000 person-years (95% CI)	Adjusted HR (95% CI)^b^	*P* value^c^
Primary CVD outcomes							
Without bariatric surgery	58 306	2401	96 557.6	24.9 (23.9-25.9)	[Reference]	[Reference]	
With bariatric surgery	28 658	549	57 228.6	9.6 (8.8-10.4)	15.3 (14.0-16.6)	0.53 (0.48-0.59)	<.001
Myocardial infarction							
Without bariatric surgery	58 343	354	96 210.2	3.7 (3.3-4.1)	[Reference]	[Reference]	
With bariatric surgery	28 621	109	57 100.2	1.9 (1.6-2.3)	1.8 (1.3-2.3)	0.80 (0.63-1.00)	.05
Heart failure							
Without bariatric surgery	58 340	1595	96 379.4	16.6 (15.8-17.4)	[Reference]	[Reference]	
With bariatric surgery	28 624	256	57 126.0	4.5 (4.0-5.1)	12.1 (11.1-13.1)	0.39 (0.34-0.45)	<.001
Ischemic stroke							
Without bariatric surgery	58 335	452	96 268.2	4.7 (4.3-5.2)	[Reference]	[Reference]	
With bariatric surgery	28 629	184	57 125.1	3.2 (2.8-3.7)	1.5 (0.8-2.1)	0.79 (0.66-0.94)	.01
Secondary CVD outcomes							
Without bariatric surgery	58 356	5424	96 150.1	56.4 (54.9-57.9)	[Reference]	[Reference]	
With bariatric surgery	28 608	1191	57 061.4	20.9 (19.7-22.1)	35.5 (33.6-37.5)	0.50 (0.46-0.53)	<.001
Secondary ischemic heart events^d^							
Without bariatric surgery	58 356	3165	96 150.1	32.9 (31.8-34.1)	[Reference]	[Reference]	
With bariatric surgery	28 608	539	57 061.4	9.4 (8.7-10.3)	23.5 (22.1-24.9)	0.38 (0.34-0.42)	<.001
Secondary cerebrovascular events^e^							
Without bariatric surgery	58 356	762	96150.1	7.9 (7.4-8.5)	[Reference]	[Reference]	
With bariatric surgery	28 608	219	57061.4	3.8 (3.4-4.4)	4.1 (3.3-4.8)	0.60 (0.51-0.70)	<.001
TIA							
Without bariatric surgery	58 356	373	96 150.1	3.9 (3.5-4.3)	[Reference]	[Reference]	
With bariatric surgery	28 608	155	57 061.4	2.7 (2.3-3.2)	1.2 (0.6-1.7)	0.72 (0.59-0.89)	.002
Atherosclerosis							
Without bariatric surgery	58 356	1007	96 150.1	10.5 (9.8-1.1)	[Reference]	[Reference]	
With bariatric surgery	28 608	246	57 061.4	4.3 (3.8-4.9)	6.2 (5.3-7.0)	0.70 (0.61-0.81)	<.001
Arterial embolism and thrombosis							
Without bariatric surgery	58 356	117	96 150.1	1.2 (1.0-1.5)	[Reference]	[Reference]	
With bariatric surgery	28 608	32	57 061.4	0.6 (0.4-0.8)	0.7 (0.4-0.9)	0.61 (0.40-0.91)	.02

Abbreviations: CKD, chronic kidney disease; CVD, cardiovascular disease; HR, hazard ratio; TIA, transient ischemic attack.

^a^ Bariatric surgery status was modeled as a time-varying covariate.

^b^ Using inverse probability of treatment weighting and adjusted for age, type of health insurance, region of residence, year of nonalcoholic fatty liver disease diagnosis, sex, smoking status, asthma, obstructive sleep apnea, obesity hypoventilation syndrome, severe urinary incontinence, chronic venous insufficiency, osteoarthritis, diabetes, hypertension, dyslipidemia, CKD, and cancer.

^c^ For the adjusted HRs comparing individuals with surgery vs without surgery status.

^d^ Angina pectoris, complications of myocardial infarction, acute coronary thrombosis, Dressler syndrome, or chronic ischemic heart diseases.

^e^ Occlusion and stenosis of precerebral or cerebral arteries not resulting in ischemic stroke, cerebral atherosclerosis, acute cerebrovascular insufficiency, or cerebral ischemia.

**Figure 2.**
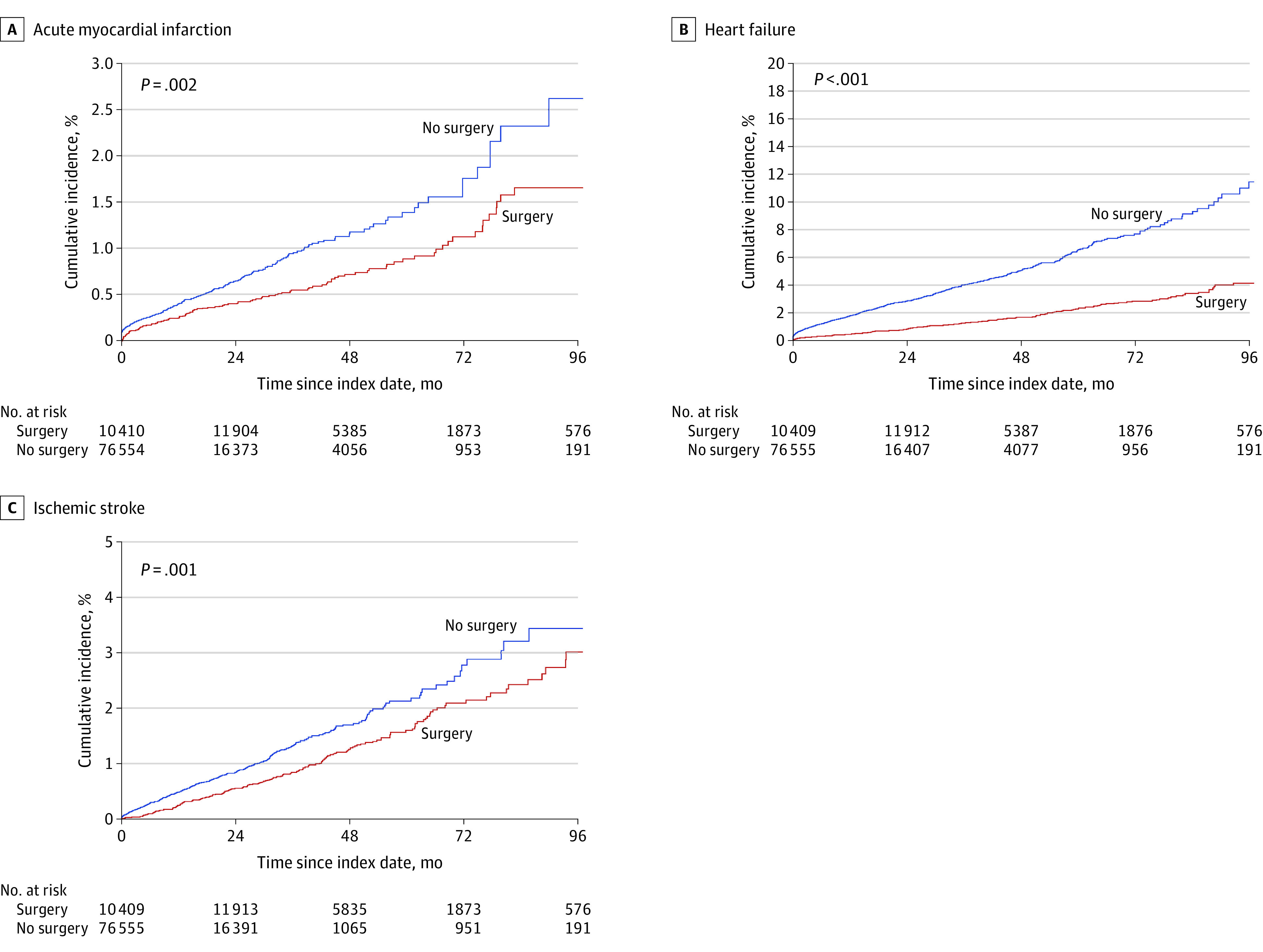
Cumulative Incidence of Myocardial Infarction, Heart Failure, and Ischemic Stroke Bariatric surgery status was modeled as a time-varying variable. Survival estimates were obtained using the Simon-Makuch method. *P* values were obtained from the Mantel and Byar test for survival comparisons of data with a time-dependent covariate.

Figure 2 and eTable 4 in the Supplement show that bariatric surgery was associated with significantly lower risks of MI, heart failure, and ischemic stroke. At 96 months, the cumulative incidence of MI was 1.7% in the surgical group vs 2.6% in the nonsurgical group, heart failure was 4.2% vs 11.5%, and ischemic stroke was 3.0% vs 3.4%. Similarly, the incidence rates for MI, heart failure, and ischemic stroke were lower in the surgical vs nonsurgical group (Table 3). Compared with those without surgery status, individuals who underwent surgery had lower adjusted hazards of MI (aHR, 0.80; 95% CI, 0.63-1.00), heart failure (aHR, 0.39; 95% CI, 0.34-0.45), and ischemic stroke (aHR, 0.79; 95% CI, 0.66-0.94).

### Secondary CVD Outcomes After Bariatric Surgery

We identified 1191 individuals in the surgical group with a secondary CVD outcome over 57 061.4 person-years and 5424 individuals in the nonsurgical group with a secondary outcome over 96 150.1 person-years (incidence rate difference, 35.5 [95% CI, 33.6-37.5] per 1000 person-years) (Table 3). The 96-month cumulative incidence of a secondary outcome was 17.3% in the surgical group and 28.2% in the nonsurgical group (Figure 1C; eTable 4 in the Supplement). Surgery status was associated with a 50% lower adjusted hazard of secondary outcomes than nonsurgical status (aHR, 0.50; 95% CI, 0.46-0.53) (Table 3).

eFigure 4 and eTable 4 in the Supplement show significantly lower incidence of all 5 components of the secondary CVD outcomes in the surgical vs the nonsurgical group. Compared with those with nonsurgical status, individuals who underwent surgery had significant hazard reductions for secondary ischemic heart events (aHR, 0.38; 95% CI, 0.34-0.42), secondary cerebrovascular events (aHR, 0.60; 95% CI, 0.51-0.70), and atherosclerosis (aHR, 0.70; 95% CI, 0.61-0.81). Comparable results were observed for the association of surgery with hazards of transient ischemic attack (aHR, 0.72; 95% CI, 0.59-0.89) and arterial embolism and thrombosis (aHR, 0.61; 95% CI, 0.40-0.91).

### Sensitivity Analyses 

Bariatric surgery remained a significant factor in lower hazard of composite CVEs, primary CVD outcomes, and secondary CVD outcomes after we limited the outcomes to those with at least 2 separate claims (eTable 5 in the Supplement). For example, surgical status was associated with 52% lower risk of composite CVEs than nonsurgical status in the redefined analysis (aHR, 0.48; 95% CI, 0.45-0.52). Similarly, the surgical group had lower hazards of composite CVEs, primary composite CVD outcome, and secondary composite CVE outcome when we (1) extended the cohort to include all participants with a BMI of 35 or higher, (2) used inverse probability of censoring weighting to control for potential informative censoring, and (3) limited the exposure to RYGB and sleeve gastrectomy (eTables 6-8 in the Supplement). For example, the risk of composite CVEs associated with receiving RYGB or sleeve gastrectomy was 49% lower than nonsurgical care (aHR, 0.51, 95% CI, 0.47-0.55). Examining the bias factor and E-value estimates and comparing them with known risk factors for CVD revealed that an unmeasured confounder was unlikely to fully explain the observed CVD risk reductions associated with bariatric surgery (eTables 9 and 10 and eMethods 3 in the Supplement).

## Discussion

Previous studies have found that bariatric surgery was associated with long-term histological improvements in NAFLD.^48,49^ However, less is known about whether bariatric surgery is associated with reduced CVD risk in individuals with severe obesity and NAFLD. In this large, population-based retrospective cohort study, individuals with severe obesity and NAFLD who underwent bariatric surgery had one-half the CVD incidence compared with individuals who received nonsurgical care. The decreased CVD risk was associated with lower incidences of both primary and secondary composite CVD outcomes.

Several studies have investigated the association between bariatric surgery and CVD risk.^32,50,51,52,53,54,55^ In a meta-analysis of adults with obesity from 39 cohort studies, bariatric surgery was associated with lower incidences of heart failure (HR, 0.59), MI (HR, 0.58), and stroke (HR, 0.64).^56^ Those results support an earlier finding from a pooled analysis of 4 observational studies in which bariatric surgery was associated with reduced risk of adverse CVE (odds ratio [OR], 0.54), MI (OR, 0.46), and stroke (OR, 0.49).^57^ Aminian et al^35^ found that bariatric surgery was associated with decreased risk of major CVEs in individuals with NASH (HR, 0.30). Results of the present study support these previous findings and extend them to the full NAFLD spectrum.

Nonalcoholic fatty liver disease and CVD share common risk factors associated with elevated cardiometabolic risk. In addition, NAFLD is an independent risk of multiple deleterious cardiovascular complications, such as cardiac arrhythmias, valvular heart disease, atherosclerosis, and cardiomyopathy.^21,58,59^ Several pathophysiological mechanisms may help explain the elevated risk of CVD in NAFLD. Nonalcoholic fatty liver disease is associated with increased ectopic hepatic fat and hepatic insulin resistance. Ectopic hepatic fat may contribute to local systemic inflammation, increased atherosclerosis, and cardiometabolic risk.^60^ Furthermore, adipose tissue releases bioactive mediators that alter coagulation, fibrinolysis, and inflammation, resulting in endothelial dysfunction and atherosclerosis.^13^

Interventions that target NAFLD-associated obesity could potentially reduce CVD risk in this patient group. However, pharmacological agents for NAFLD are currently not available, and the benefits of lifestyle modifications are difficult to sustain.^61^ Furthermore, a pharmaceutical intervention for NAFLD needs to provide substantial clinical benefits at a modest annual price to be cost-effective.^62^ The weight loss attendant with bariatric surgery is associated with improved overall CVD risk profile and NAFLD surrogates, including fibrosis and cirrhosis.^30,61,63,64,65^ Such improvements may contribute to the observed CVD risk attenuations in individuals with NAFLD who underwent surgery, especially the substantial decrease in heart failure incidence.^34,66,67,68,69^ In addition, bariatric surgery is a cost-effective intervention for individuals with overweight or obesity and NASH-related cirrhosis, and it is associated with reduced cancer risk in this group.^70,71^

### Strengths and Limitations

This study has several strengths. To our knowledge, this study was the first to examine the association of bariatric surgery with CVD risk in the full NAFLD spectrum. It also had a large sample size with individual-level claims data and a retrospective cohort study design that may have mitigated the impact of surveillance bias. The sample included 10 404 individuals with NAFLD who underwent sleeve gastrectomy, the most frequently performed bariatric surgery in the US. We used a causal inference approach to adjust for any potential confounding by indication, with surgery status modeled as time varying to address immortal time bias. We also conducted multiple sensitivity analyses to ensure the robustness of the main findings.

This study also has several limitations. The use of claims data and observational study design might leave room for unmeasured confounding. Based on the E-value analysis, the observed CVD risk reduction could be fully explained by an unmeasured confounder with an HR of 2.56 and association with both bariatric surgery and CVD in addition to the confounders included in the analysis. We believe that this magnitude of confounding is unlikely to remain unmeasured given the variables included in the adjusted analyses. Although claims data may have some misclassifications, the main findings did not change when we redefined CVD incidence to at least 2 claims records.

The mean follow-up time was 21.1 months, with a high censoring rate in the later years of the study and lack of data on out-of-hospital mortality. However, the findings were consistent with the results obtained from the inverse probability of censoring weighting analysis. The cohort was limited to individuals aged 18 to 64 years, and we could not adjust for race and ethnicity because the data were not included in the MarketScan database. Nevertheless, the cohort profile was consistent with that in the study by Campos et al,^72^ which analyzed the characteristics of nearly 2 million individuals who underwent bariatric surgery from 1993 to 2016, suggesting that the sample in the present study resembles those who underwent bariatric surgery in the US. We could not ascertain the association between surgery and CVD by disease phenotype because of the lack of reliable noninvasive diagnostic tools for NAFLD. However, all individuals had severe obesity, and the proportion of cirrhosis was balanced between the surgical and nonsurgical groups. Furthermore, the results were consistent when we extended the sample to include those with a BMI of 35 or higher.

## Conclusions

In this cohort study, adults with severe obesity and NAFLD who underwent bariatric surgery appeared to have a lower CVD risk than those who received nonsurgical care. The findings provide evidence in support of bariatric surgery as an effective therapeutic tool to lower elevated CVD risk for select individuals with obesity and NAFLD. Although bariatric surgery is a more aggressive approach than lifestyle modifications, it may be associated with other benefits, such as improved quality of life and decreased long-term health care burden.

## Notes

Supplement. **eMethods 1.** General Statistical Guidance **eTable 1.** Diagnostic Codes for Inclusion and Exclusion Criteria Used to Identify Nonalcoholic Fatty Liver Disease **eTable 2.** Diagnosis and Procedure Codes for Bariatric Surgery, Study Outcomes, and Covariates **eFigure 1.** Study Design Outlining Bariatric Surgery Status Modeled as Time-Varying **eFigure 2.** Study Cohort Inclusion and Exclusion Flow Diagram **eFigure 3.** Annual Distributions of Bariatric Surgeries Performed in a Cohort of Commercially Insured Adults with Nonalcoholic Fatty Liver Disease and Severe Obesity from 2008-2017 **eTable 3.** Propensity Score Weighted Baseline Characteristics of the Study Sample by Bariatric Surgery Status, Adults with Nonalcoholic Fatty Liver Disease and Severe Obesity, 2008-2017 (n=86,964) **eTable 4.** Crude and Inverse Probability of Treatment Adjusted Cumulative Incidence (%) and 95% Confidence Intervals Stratified by Bariatric Surgery Status for Cardiovascular Outcomes at Two, Four, Six, and Eight Years After the First Nonalcoholic Fatty Liver Disease (NAFLD) Diagnosis, Severely Obese NAFLD Adults, 2008 to 2017 (n=86,964) **eFigure 4.** Cumulative Incidence of A) Ischemic Heart Event B) Transient Ischemic Attack C) Cerebrovascular Event D) Atherosclerosis E) Arterial Embolism and Thrombosis **eMethods 2.** Sensitivity Analyses **eTable 5.** Associations Between Bariatric Surgery and Risk of Cardiovascular Disease Outcomes in Adults With Nonalcoholic Fatty Liver Disease and Severe Obesity, 2008-2017 (n=86,964) **eTable 6.** Associations Between Bariatric Surgery and Risk of Cardiovascular Disease Outcomes in Overweight Adults With Nonalcoholic Fatty Liver Disease, 2008-2017 (n=123,341) **eTable 7.** Inverse Probability of Censoring Weight Adjusted Associations Between Bariatric Surgery and Risk of Cardiovascular Outcomes in Overweight Adults With Nonalcoholic Fatty Liver Disease, 2008-2017 (n=86,964) **eTable 8.** Associations Between Roux-en-Y Gastric Bypass and Sleeve Gastrectomy Bariatric Surgeries and Risk of Cardiovascular Disease Outcomes in Adults With Nonalcoholic Fatty Liver Disease and Severe Obesity, 2008-2017 (n=74,831) **eMethods 3.** The E-value and Bias Factors Sensitivity Analyses for Unmeasured Confounding **eTable 9.** Corrected Estimates and Confidence Intervals for Unmeasured Confounding for the Effect of Bariatric Surgery on the Risk of Cardiovascular Outcomes in Severely Obese Adults With Nonalcoholic Fatty Liver Disease, 2008-2017 (n=86,964) **eTable 10.** Point Estimates Upper-Level Confidence Intervals and E-Values for the Effect of Bariatric Surgery on the Risk of Cardiovascular Outcomes in Severely Obese Adults With Nonalcoholic Fatty Liver Disease, 2008-2017 (n=86,964)
eReferences
