# Supplementary material for: Association of Bariatric Surgery With Cardiovascular Outcomes in Adults With Severe Obesity and Nonalcoholic Fatty Liver Disease
Source: JAMA Netw Open. 2022 Oct 7;5(10):e2235003. doi: 10.1001/jamanetworkopen.2022.35003 (PMC9547320; doi:10.1001/jamanetworkopen.2022.35003)
Supplement: Supplement. — eMethods 1. General Statistical Guidance eTable 1. Diagnostic Codes for Inclusion and Exclusion Criteria Used to Identify Nonalcoholic Fatty Liver Disease eTable 2. Diagnosis and Procedure Codes for Bariatric Surgery, Study Outcomes, and Covariates eFigure 1. Study Design Outlining Bariatric Surgery Status Modeled as Time-Varying eFigure 2. Study Cohort Inclusion and Exclusion Flow Diagram eFigure 3. Annual Distributions of Bariatric Surgeries Performed in a Cohort of Commercially Insured Adults with Nonalcoholic Fatty Liver Disease and Severe Obesity from 2008-2017 eTable 3. Propensity Score Weighted Baseline Characteristics of the Study Sample by Bariatric Surgery Status, Adults with Nonalcoholic Fatty Liver Disease and Severe Obesity, 2008-2017 (n=86,964) eTable 4. Crude and Inverse Probability of Treatment Adjusted Cumulative Incidence (%) and 95% Confidence Intervals Stratified by Bariatric Surgery Status for Cardiovascular Outcomes at Two, Four, Six, and Eight Years After the First Nonalcoholic Fatty Liver Disease (NAFLD) Diagnosis, Severely Obese NAFLD Adults, 2008 to 2017 (n=86,964) eFigure 4. Cumulative Incidence of A) Ischemic Heart Event B) Transient Ischemic Attack C) Cerebrovascular Event D) Atherosclerosis E) Arterial Embolism and Thrombosis eMethods 2. Sensitivity Analyses eTable 5. Associations Between Bariatric Surgery and Risk of Cardiovascular Disease Outcomes in Adults With Nonalcoholic Fatty Liver Disease and Severe Obesity, 2008-2017 (n=86,964) eTable 6. Associations Between Bariatric Surgery and Risk of Cardiovascular Disease Outcomes in Overweight Adults With Nonalcoholic Fatty Liver Disease, 2008-2017 (n=123,341) eTable 7. Inverse Probability of Censoring Weight Adjusted Associations Between Bariatric Surgery and Risk of Cardiovascular Outcomes in Overweight Adults With Nonalcoholic Fatty Liver Disease, 2008-2017 (n=86,964) eTable 8. Associations Between Roux-en-Y Gastric Bypass and Sleeve Gastrectomy Bariatric Surgeries and Risk of Cardiova [file jamanetwopen-e2235003-s001.pdf]

Supplemental Online Content

Elsaid MI, Li Y, Bridges JFP, Brock G, Minacapelli CD, Rustgi VK. Association of bariatric surgery with cardiovascular outcomes in adults with severe obesity and nonalcoholic fatty liver disease. *JAMA Netw Open*. 2022;5(10):e2235003. doi:10.1001/jamanetworkopen.2022.35003

eMethods 1. General Statistical Guidance

eTable 1. Diagnostic Codes for Inclusion and Exclusion Criteria Used to Identify Nonalcoholic Fatty Liver Disease

eTable 2. Diagnosis and Procedure Codes for Bariatric Surgery, Study Outcomes, and Covariates

eFigure 1. Study Design Outlining Bariatric Surgery Status Modeled as Time-Varying

eFigure 2. Study Cohort Inclusion and Exclusion Flow Diagram

eFigure 3. Annual Distributions of Bariatric Surgeries Performed in a Cohort of Commercially Insured Adults with Nonalcoholic Fatty Liver Disease and Severe Obesity from 2008-2017

eTable 3. Propensity Score Weighted Baseline Characteristics of the Study Sample by Bariatric Surgery Status, Adults with Nonalcoholic Fatty Liver Disease and Severe Obesity, 2008-2017 (n=86,964)

eTable 4. Crude and Inverse Probability of Treatment Adjusted Cumulative Incidence (%) and 95% Confidence Intervals Stratified by Bariatric Surgery Status for Cardiovascular Outcomes at Two, Four, Six, and Eight Years After the First Nonalcoholic Fatty Liver Disease (NAFLD) Diagnosis, Severely Obese NAFLD Adults, 2008 to 2017 (n=86,964)

eFigure 4. Cumulative Incidence of A) Ischemic Heart Event B) Transient Ischemic Attack C) Cerebrovascular Event D) Atherosclerosis E) Arterial Embolism and Thrombosis

eMethods 2. Sensitivity Analyses

© 2022 Elsaid MI et al. *JAMA Network Open.*

eTable 5. Associations Between Bariatric Surgery and Risk of Cardiovascular Disease Outcomes in Adults With Nonalcoholic Fatty Liver Disease and Severe Obesity, 2008-2017 (n=86,964)

eTable 6. Associations Between Bariatric Surgery and Risk of Cardiovascular Disease Outcomes in Overweight Adults With Nonalcoholic Fatty Liver Disease, 2008-2017 (n=123,341)

eTable 7. Inverse Probability of Censoring Weight Adjusted Associations Between Bariatric Surgery and Risk of Cardiovascular Outcomes in Overweight Adults With Nonalcoholic Fatty Liver Disease, 2008-2017 (n=86,964)

eTable 8. Associations Between Roux-en-Y Gastric Bypass and Sleeve Gastrectomy Bariatric Surgeries and Risk of Cardiovascular Disease Outcomes in Adults With Nonalcoholic Fatty Liver Disease and Severe Obesity, 2008-2017 (n=74,831)

eMethods 3. The E-value and Bias Factors Sensitivity Analyses for Unmeasured Confounding

eTable 9. Corrected Estimates and Confidence Intervals for Unmeasured Confounding for the Effect of Bariatric Surgery on the Risk of Cardiovascular Outcomes in Severely Obese Adults With Nonalcoholic Fatty Liver Disease, 2008-2017 (n=86,964)

eTable 10. Point Estimates Upper-Level Confidence Intervals and E-Values for the Effect of Bariatric Surgery on the Risk of Cardiovascular Outcomes in Severely Obese Adults With Nonalcoholic Fatty Liver Disease, 2008-2017 (n=86,964)

eReferences

This supplemental material has been provided by the authors to give readers additional information about their work.

© 2022 Elsaid MI et al. *JAMA Network Open.*

eMethods 1. General Statistical Guidance

Missing Values

The analytic sample had complete data on all included demographics (age, gender, type of insurance, region of residence etc.). Nevertheless, under-reporting could result in missing data related to smoking status and medical history variables. For example, a participant without a diagnostic code for hypertension will be classified as without hypertension. However, the absence of diagnostic code could be due to either a true negative or false negative (patients had hypertension but was not coded as such). Consistent with prior studies, we limited our analysis to participants with at least one year of continuous enrollment prior to the first date of nonalcoholic fatty liver disease (NAFLD) diagnosis to ensure accurate reporting of patients' medical history in both the exposed and unexposed groups (1-3). As such, the presence of any missing data will result in non-differential misclassification which would only bias our findings towards the null value of no association between bariatric surgery and reductions in the risk of cardiovascular disease.

Severe Obesity Sample Restriction

We restricted the study sample to severely obese patients to reflect on both the clinical guidelines implemented during the study period and the nature of the administrative data used in the analysis. Both the 2008 and 2013 clinical guidelines by the American Association of Clinical Endocrinologists, the Obesity Society, and the American Society for Metabolic & Bariatric Surgery (ASMBS) suggest that bariatric surgery should be offered to 1) individuals with BMI ≥40 kg/m^2 with or without coexisting comorbidities, and 2) patients with both BMI ≥35 kg/m^2 and one or more severe obesity-related co-morbidities (4, 5). Several health insurance providers do not include NAFLD/NASH as a qualifying condition for surgery coverage for those with BMI ≥35 kg/m^2. In those NAFLD patients with BMI ≥35 kg/m^2, the probability of receiving surgery is conditioned on the provider's policy. We conducted a sensitivity analysis to examine the impacts of extending the study sample to NAFLD patients with BMI ≥35 kg/m^2.

© 2022 Elsaid MI et al. *JAMA Network Open.*

eTable 1. Diagnostic Codes for Inclusion and Exclusion Criteria Used to Identify Nonalcoholic Fatty Liver Disease

**Inclusion Criteria**	ICD-9: 571.5, 571.8, 571.9<br>ICD-10: K74.0, K74.1, K74.2, K74.60, K74.69, K75.81, K76.0
**Exclusion Criteria**	
Alcoholic liver disease	ICD-9: 571.0, 571.1, 571.2, 571.3<br>ICD-10: K70.x
Alcohol use disorder (excluding liver related)	ICD-9: 291.0, 291.1, 291.2, 291.3, 291.4, 291.5, 291.8, 291.81, 291.82, 291.89, 291.9, 303.00, 303.01, 303.02, 303.03, 303.90, 303.91, 303.92, 303.93, 305.00, 305.01, 305.02, 305.03, 357.5, 425.5, 535.30, 535.31, E860.0<br>ICD-10: F10.1x, F10.2x, F10.9x, G62.1, G31.2, G72.1, I42.6, K29.20, K29.21, O99310, O99311, O99312, O99313, O99314, O99315
Biliary cirrhosis	ICD-9: 571.6<br>ICD-10: K74.3, K74.4, K74.5
Hepatitis (viral and autoimmune)	ICD-9: 070.0, 070.1, 070.20, 070.21, 070.22, 070.32, 070.23, 070.33, 070.30, 070.31, 070.41, 070.42, 070.52, 070.43, 070.53, 070.44, 070.54, 070.49, 070.59, 070.51, 070.6, 070.70, 070.71, 070.9, V02.60, V02.61, V02.62, V02.69, 571.40, 571.41, 571.42, 571.49, 573.1, 573.2<br>ICD-10: B15.0, B15.9, B16.2, B19.11, B16.0, B18.1, B18.0, B16.9, B19.10, B16.1, B17.11, B17.0, B17.2, B18.2, B17.8, B18.8, B18.9, B17.10, B19.0, B19.20, B19.21, B19.9, Z22.50, Z22.51, Z22.52, Z22.59, K73.9, K73.0, K75.4, K73.2, K73.8, K77

© 2022 Elsaid MI et al. *JAMA Network Open.*

eTable 2. Diagnosis and Procedure Codes for Bariatric Surgery, Study Outcomes, and Covariates

Open Roux-en-Y gastric bypass	CPT: 43846, 43847, 43633
	ICD-9 Procedure Code: 44.39, 43.7
	ICD-10 Procedure Code: 0D16078, 0DB60ZZ
Open sleeve gastrectomy/ vertical-banded gastroplasty	CPT: 43842, 43843
	ICD-9 Procedure Code: 44.68, 44.69
	ICD-10 Procedure Code: 0DQ64ZZ, 0DQ60ZZ
Laparoscopic adjustable gastric band	CPT: 43770
	ICD-9 Procedure Code: 44.95
	ICD-10 Procedure Code: 0DV64CZ
Biliopancreatic diversion/duodenal switch (BPD-DS)	CPT: 43845
	ICD-9 Procedure Code: 45.91, 43.89, 45.51
	ICD-10 Procedure Code: 0D190Z9, 0DB60ZZ, 0DB80ZZ
Laparoscopic sleeve gastrectomy	CPT: 43775
	ICD-9 Procedure Code: 43.89, 43.82
	ICD-10 Procedure Code: 0DB60ZZ
Laparoscopic Roux-en-Y gastric bypass	CPT: 43644, 43645
	ICD-9 Procedure Code: 44.38
	ICD-10 Procedure Code: 0D16479, 0D1647A, 0D164J9, 0D164JA, 0D164K9, 0D164KA
Open and other partial gastrectomy	CPT: 43659
	ICD-9 Procedure Code: 43.89
	ICD-10 Procedure Code: 0DB60ZZ
Secondary Ischemic heart events	ICD-9: 411.X, 413.X, 414.X, 429.2
	ICD-10: I20.X, I23.X-I25.X (except I25.2)
Myocardial infarction	ICD-9: 410.X, 410.XX
	ICD-10: I21.X–I22.X
Heart failure	ICD-9: 428.X, 428XX
	ICD-10: I50.X
Ischemic stroke	ICD-9: 433.X1, 434.X1
	ICD-10: I63.X, I63XX, I63XXX, I69.3X
Atherosclerosis	ICD-9: 440.X
	ICD-10: I70.X
Transient ischemic Attack	ICD-9: 435.3,435.0, 435.1, 435.2, 435.8, 435.9
	ICD-10: G45.0, G45.8, G45.1, G45.2, G45.9, I67.841, I67.848
Arterial Embolism and Thrombosis	ICD-9: 444.X
	ICD-10: I74.X
Cerebrovascular Event	ICD-9: 433.00, 433.10, 433.20, 433.30, 433.80, 433.90, 434.00, 434.10, 434.90, 437.0, 437.1
	ICD-10: I65.X, I66.X, I67.2, I67.81, I67.82, I67.89

© 2022 Elsaid MI et al. *JAMA Network Open.*

eTable 2 Continued. Diagnosis and Procedure Codes for Bariatric Surgery, Study Outcomes, and Covariates

Cancer	ICD-9: 140.xx-195.xx, 200.xx-208.xx, 233.0
	ICD-10: C00–C76, C90.0X
Morbid obesity	ICD-9: 278.01
	ICD-10: E66.01
BMI 40.0 – 44.9, adult	ICD-9: V85.41
	ICD-10: Z68.41
BMI 45.0 – 49.9, adult	ICD-9: V85.42
	ICD-10: Z68.42
BMI 50.0 – 59.9, adult	ICD-9: V85.43
	ICD-10: Z68.43
BMI 60.0 – 69.9, adult	ICD-9: V85.44
	ICD-10: Z68.44
BMI 70 and over, adult	ICD-9: V85.45
	ICD-10: Z68.45
Smoking	ICD-9: 305.1, 649.0x, 989.84, V15.82
	ICD-10: F17.X, T65.2X, Z71.6, Z72.0, Z87.891
	CPT: 99406, 99407, G0436, G0437, G9016, S9453, S4995, G9276, G9458, 1034F, 4004F, 4001F
Asthma	ICD-9: 493.00, 493.01, 493.02, 493.10, 493.11, 493.12, 493.20, 493.21, 493.22, 493.81, 493.82, 493.90, 493.91, 493.92
	ICD-10: J45.20, J45.21, J45.22, J45.30, J45.31, J45.32, J45.40, J45.41, J45.42, J45.50, J45.51, J45.52, J45.90, J45.901, J45.902, J45.909, J45.990, J45.991, J45.998
Obstructive sleep apnea	ICD-9: 327.23
	ICD-10: G47.33
Obesity hypoventilation syndrome	ICD-9: 278.03
	ICD-10: E66.2
Osteoarthritis	ICD-9: 715.x
	ICD-10: M15.X, M16.X, M17.X, M18.X, M19.X
Diabetes	ICD-9: 250.X
	ICD-10: E08.X, E09.X, E10.X, E11.X, E13.X
Hypertension	ICD-9: 401.X, 402.X, 403.X, 404.X, 405.X
	ICD-10: I10, I15.x
Dyslipidemia	ICD-9: 272.X
Chronic kidney disease	ICD-9: 585.x
	ICD-10: N18, N18.1, N18.2, N18.3, N18.4, N18.5, N18.6, N18.9
Severe urinary incontinence	ICD-9: 788.30, 625.6, 788.32, 788.31, 788.34, 788.35, 788.37, 788.33, 788.36, 599.89
	ICD-10: R32, N39.3, N39.41-N39.46, N39.8

© 2022 Elsaid MI et al. *JAMA Network Open.*

Chronic venous insufficiency	ICD-9: 459.81
	ICD-10: I87.2

© 2022 Elsaid MI et al. *JAMA Network Open.*

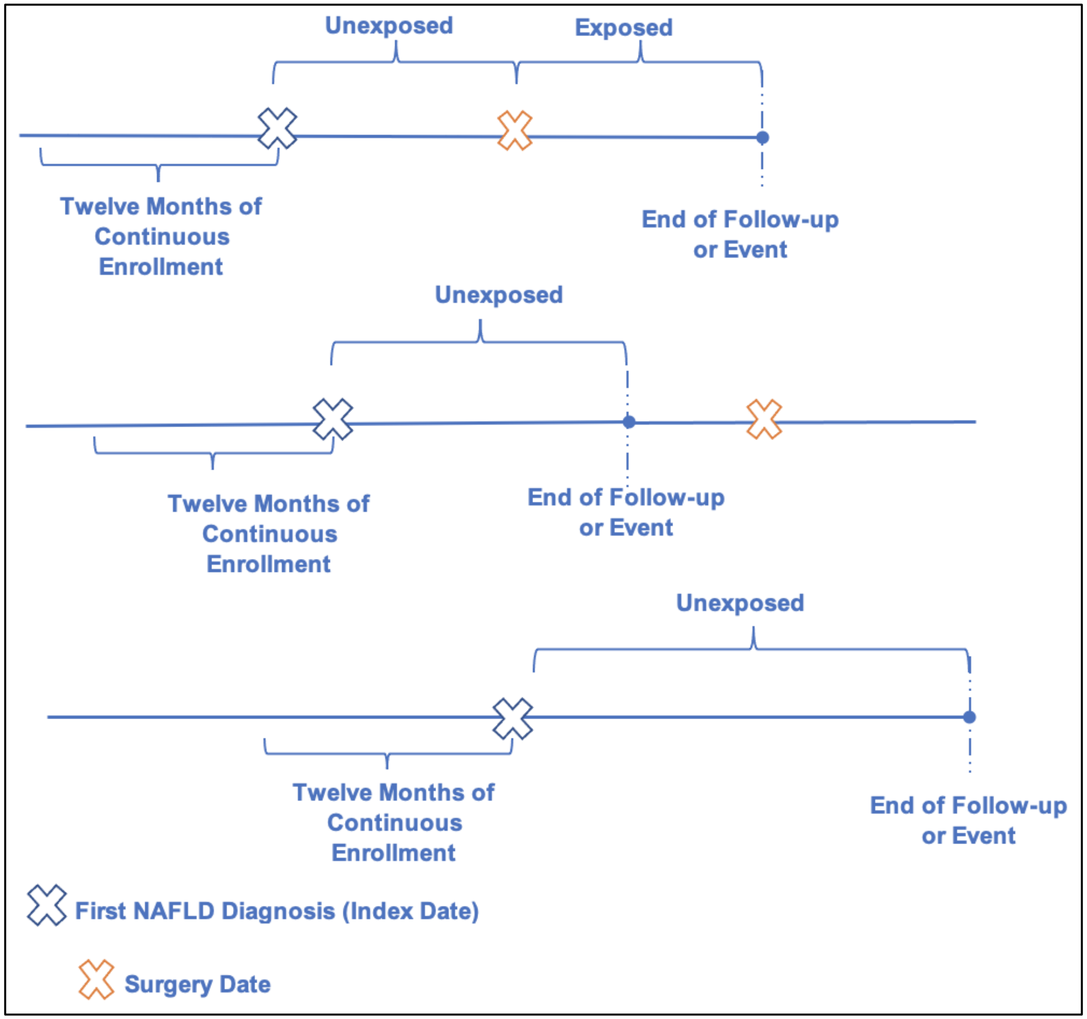

Exposed= classified as a surgical patient
Unexposed = Classified as a nonsurgical patient

eFigure 1. Study Design Outlining Bariatric Surgery Status Modeled as Time-Varying

© 2022 Elsaid MI et al. *JAMA Network Open.*

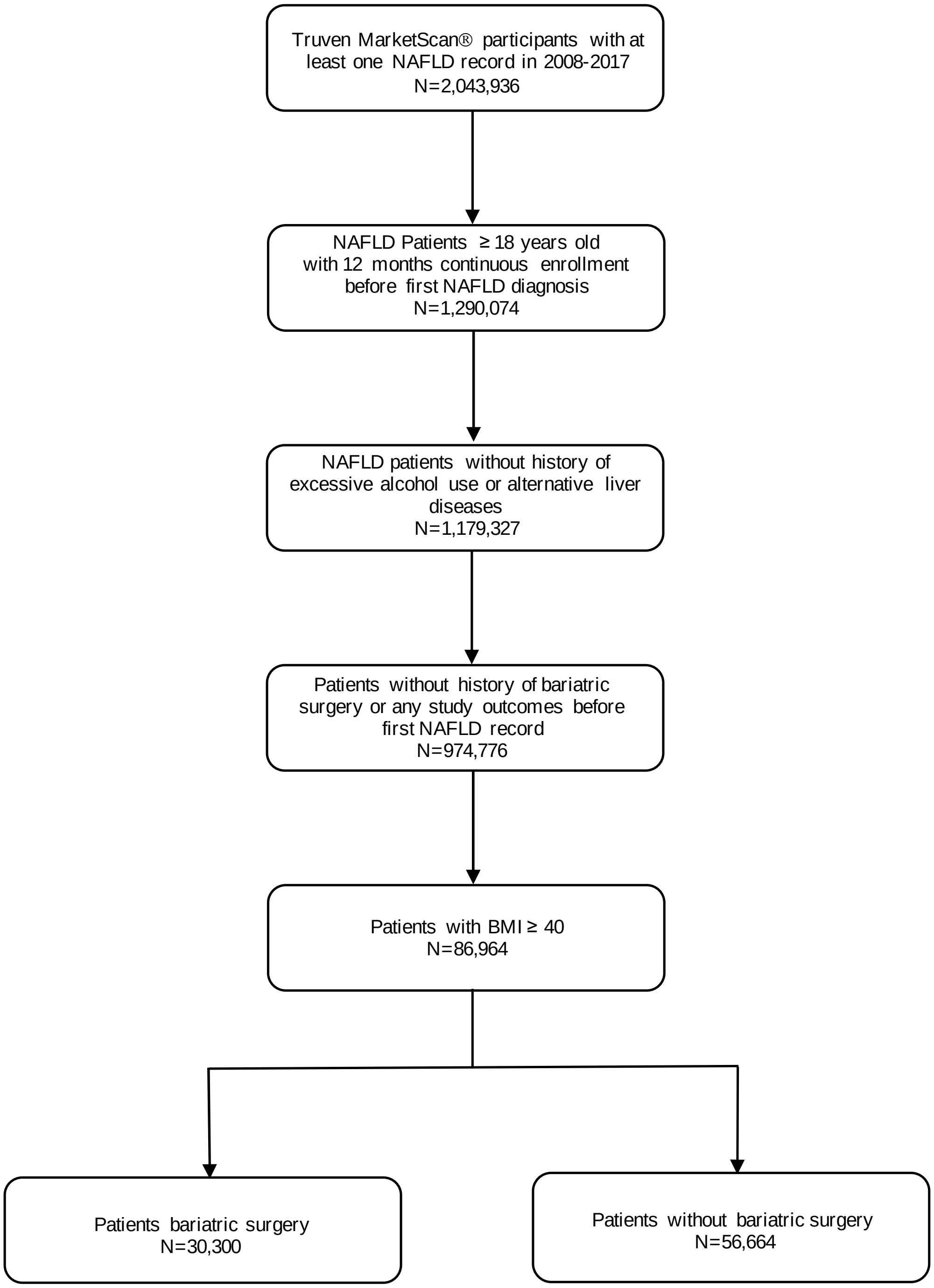

* NAFLD = Nonalcoholic fatty liver disease; BMI = body mass index

eFigure 2. Study Cohort Inclusion and Exclusion Flow Diagram

© 2022 Elsaid MI et al. *JAMA Network Open.*

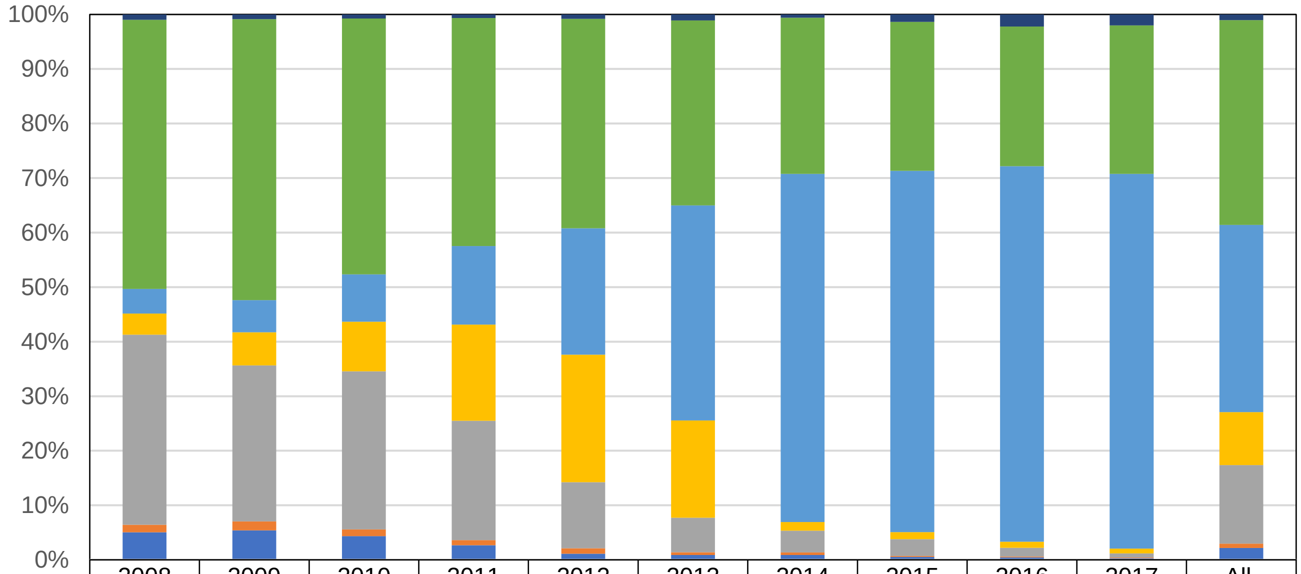

	2008	2009	2010	2011	2012	2013	2014	2015	2016	2017	All
Open and other Partial Gastrectomy	15	30	27	25	33	37	21	36	55	39	318
Lap RYGB	753	1,783	1,674	1,568	1,567	1,150	992	719	639	526	11,371
Lap SG	69	205	310	540	945	1,337	2,211	1,743	1,717	1,327	10,404
BPS-DS	59	210	324	661	954	604	55	34	28	17	2,946
Lap AGB	532	990	1,035	821	495	215	138	81	40	15	4,362
Open VBG/SG	21	57	44	36	40	17	16	5	4	2	242
Open RYGB	77	187	155	100	46	30	31	14	11	6	657

RYGB, Roux-en-Y gastric bypass; lap, laparoscopic; SG, sleeve gastrectomy; VBG, vertical-banded gastroplasty; AGB, adjustable gastric band; BPS-DS, biliopancreatic diversion/duodenal switch; other, other partial gastrectomy

eFigure 3. Annual Distributions of Bariatric Surgeries Performed in a Cohort of Commercially Insured Adults with Nonalcoholic Fatty Liver Disease and Severe Obesity from 2008-2017

© 2022 Elsaid MI et al. *JAMA Network Open.*

eTable 3. Propensity Score Weighted Baseline Characteristics of the Study Sample by Bariatric Surgery Status, Adults with Nonalcoholic Fatty Liver Disease and Severe Obesity, 2008-2017 (n=86,964)

Patient Characteristics	No Bariatric Surgery Weighted %[§]	Bariatric Surgery Weighted %[§]	Unweighted P-value[c]	Weighted P-value[d]
**Patient Demographics[a]**				
**Age**, years			<0.001	0.15
Mean (SD)	44.38 (10.39)	44.27 (9.95)		
Median (25th, 75th)	45.0 (37.0, 53.0)	45.0 (37.0, 53.0)		
**Age Group**, n (%)			<0.001	0.93
18-34	20.17	20.02		
35-44	28.27	28.35		
45-54	31.24	31.41		
55+	20.31	20.23		
**Gender**, **n (%)**			<0.001	0.47
Male	31.28	31.02		
Female	68.72	68.98		
**Region of Residence**, n (%)			<0.001	0.92
Northeast	18.43	18.59		
North Central	18.75	18.50		
South	43.66	43.69		
West	17.45	17.48		
Unknown	1.71	1.74		
**Type of Health Insurance**, n (%)			<0.001	0.67
Preferred Provider Organization	61.74	61.72		
Health maintenance organization	11.54	11.32		
Comprehensive	1.87	1.88		
Point-of- Service with Capitation	8.13	8.00		
Other	16.72	17.07		
**Year of NAFLD Diagnosis**, n (%)			<0.001	0.83
2008	3.03	3.05		
2009	7.82	7.88		
2010	8.27	8.40		
2011	9.34	9.49		
2012	11.85	12.04		
2013	9.87	9.92		
2014	12.31	12.37		
2015	10.71	10.68		
2016	13.19	13.05		
2017	13.61	13.12		
**History of Smoking**, n (%)	8.29	8.32	<0.001	0.90
**Medical History[b], ** n (%)				
**Asthma,** n (%)	13.72	14.06	0.04	0.18
**Obstructive Sleep Apnea**, n (%)	27.69	28.02	<0.001	0.32
**Obesity Hypoventilation Syndrome**, n (%)	0.40	0.41	0.23	0.89
**Severe Urinary Incontinence**, n (%)	3.36	3.41	<0.001	0.70
**Chronic Venous Insufficiency**, n (%)	1.87	1.90	0.02	0.76
**Osteoarthritis,** n (%)	16.37	16.69	0.23	0.26
**Diabetes**, n (%)	33.39	33.60	0.01	0.56
**Hypertension**, n (%)	57.14	57.44	<0.001	0.42
**Dyslipidemia**, n (%)	45.68	46.07	0.09	0.30
**Chronic Kidney Disease**, n (%)	1.75	1.79	<0.001	0.69

© 2022 Elsaid MI et al. *JAMA Network Open.*

**Cancer**, n (%)	11.38	11.39	<0.001	0.97
**Cirrhosis**, n (%)	2.38	2.66	0.002	0.01

[a] Obtained on the NAFLD index date

[b] Obtained from the twelve months preceding the NAFLD index date

[c] surgery versus non-surgical chi-square tests for categorical variables and Student t tests for continuous variables

[d] Inverse probability of treatment-weighted chi-square test for categorical variables and Student t tests for continuous variables for the differences between surgery versus non-surgical groups. All patient demographics and medical history variables were used to estimate the weights

© 2022 Elsaid MI et al. *JAMA Network Open.*

eTable 4. Crude and Inverse Probability of Treatment Adjusted Cumulative Incidence (%) and 95% Confidence Intervals Stratified by Bariatric Surgery Status for Cardiovascular Outcomes at Two, Four, Six, and Eight Years After the First Nonalcoholic Fatty Liver Disease (NAFLD) Diagnosis, Severely Obese NAFLD Adults, 2008 to 2017 (n=86,964)

Outcomes		Years Since First NAFLD Diagnosis						
	Two		Four		Six		Eight	
	Crude	Adjusted[d]	Crude	Adjusted[d]	Crude	Adjusted[d]	Crude	Adjusted[d]
**Any Cardiovascular Event**								
No Bariatric Surgery	12.78 (12.46 to 13.12)	13.39 (13.03 to 13.76)	21.13 (20.50 to 21.78)	23.70 (22.99 to 24.49)	28.18 (27.04 to 29.35)	33.96 (32.62 to 35.35)	35.61 (33.35 to 37.98)	46.30 (43.63 to 49.14)
Bariatric Surgery	5.04 (4.71 to 5.40)	6.82 (6.45 to 7.21)	10.39 (9.83 to 10.99)	12.07 (11.43 to 12.75)	15.56 (14.68 to 16.49)	17.29 (16.32 to 18.32)	21.57 (20.09 to 23.15)	23.58 (21.97 to 25.31)
**Primary Outcomes**								
No Bariatric Surgery	4.30 (4.10 to 4.51)	4.28 (4.08 to 4.49)	7.76 (7.33 to 8.21)	8.04 (7.62 to 8.48)	11.80 (10.88 to 12.78)	12.82 (11.96 to 13.75)	16.69 (14.65 to 18.98)	18.28 (16.54 to 20.21)
Bariatric Surgery	1.78 (1.58 to 2.01)	2.28 (2.08 to 2.50)	3.62 (3.27 to 3.99)	4.28 (3.90 to 4.68)	5.94 (5.35 to 6.59)	6.82 (6.19 to 7.51)	8.55 (7.52 to 9.72)	9.73 (8.65 to 10.94)
**Myocardial Infarction**								
No Bariatric Surgery	0.65 (0.57 to 0.73)	0.61 (0.54 to 0.69)	1.18 (1.01 to 1.37)	1.11 (0.97 to 1.28)	1.76 (1.39 to 2.23)	1.80 (1.49 to 2.17)	2.62 (1.85 to 3.71)	2.48 (1.97 to 3.13)
Bariatric Surgery	0.40 (0.31 to 0.51)	0.48 (0.39 to 0.60)	0.72 (0.57 to 0.90)	0.88 (0.72 to 1.08)	1.13 (0.88 to 1.44)	1.43 (1.14 to 1.80)	1.66 (1.25 to 2.19)	1.97 (1.53 to 2.55)
**Heart failure**								
No Bariatric Surgery	2.87 (2.70 to 3.04)	2.84 (2.68 to 3.01)	5.07 (4.72 to 5.44)	5.14 (4.81 to 5.50)	7.70 (6.97 to 8.50)	8.10 (7.42 to 8.84)	11.46 (9.61 to 13.63)	11.99 (10.47 to 13.73)
Bariatric Surgery	0.84 (0.70 to 1.00)	1.11 (0.97 to 1.26)	1.68 (1.45 to 1.95)	2.00 (1.76 to 2.28)	2.85 (2.44 to 3.33)	3.15 (2.75 to 3.62)	4.15 (3.42 to 5.02)	4.67 (3.95 to 5.52)
**Ischemic Stroke**								
No Bariatric Surgery	0.84 (0.75 to 0.94)	0.85 (0.76 to 0.94)	1.70 (1.49 to 1.93)	1.78 (1.59 to 2.01)	2.77 (2.29 to 3.36)	2.90 (2.51 to 3.36)	3.43 (2.68 to 4.40)	3.91 (3.21 to 4.75)
Bariatric Surgery	0.56 (0.45 to 0.69)	0.67 (0.56 to 0.79)	1.26 (1.06 to 1.50)	1.41 (1.20 to 1.66)	2.09 (1.74 to 2.51)	2.29 (1.92 to 2.73)	3.02 (2.38 to 3.81)	3.09 (2.50 to 3.81)
**Secondary Outcomes**								
No Bariatric Surgery	9.82 (9.52 to 10.12)	10.11 (9.80 to 10.43)	16.66 (16.07 to 17.26)	18.23 (17.60 to 18.89)	22.30 (21.23 to 23.42)	25.94 (24.77 to 27.17)	28.22 (26.06 to 30.53)	35.18 (32.85 to 37.67)
Bariatric Surgery	3.67 (3.38 to 3.98)	5.02 (4.71 to 5.36)	8.01 (7.51 to 8.55)	9.06 (8.51 to 9.65)	12.26 (11.45 to 13.13)	12.90 (12.06 to 13.79)	17.26 (15.87 to 18.76)	17.49 (16.11 to 18.98)
**Secondary Ischemic Heart Event[b]**								
No Bariatric Surgery	5.79 (5.56 to 6.03)	5.84 (5.61 to 6.09)	10.03 (9.55 to 10.53)	10.72 (10.22 to 11.24)	13.54 (12.64 to 14.5)	14.87 (13.97 to 15.82)	17.67 (15.71 to 19.84)	20.51 (18.61 to 22.59)
Bariatric Surgery	1.69 (1.50 to 1.91)	2.21 (2.01 to 2.43)	3.91 (3.54 to 4.31)	4.05 (3.69 to 4.45)	5.70 (5.14 to 6.33)	5.62 (5.09 to 6.20)	8.43 (7.38 to 9.63)	7.75 (6.87 to 8.75)
**Secondary Cerebrovascular Event[c]**								
No Bariatric Surgery	1.50 (1.37 to 1.63)	1.47 (1.36 to 1.60)	2.56 (2.32 to 2.83)	2.67 (1.36 to 1.60)	3.64 (3.14 to 4.21)	3.95 (3.51 to 4.44)	4.61 (3.66 to 5.79)	5.15 (4.37 to 6.06)
Bariatric Surgery	0.62 (0.50 to 0.76)	0.88 (0.76 to 1.03)	1.46 (1.25 to 1.71)	1.60 (1.38 to 1.84)	2.51 (2.13 to 2.97)	2.36 (2.02 to 2.77)	3.50 (2.87 to 4.27)	3.08 (2.55 to 3.72)
**Transient Ischemic Attack**								
No Bariatric Surgery	0.76 (0.67 to 0.86)	0.75 (0.67 to 0.84)	1.43 (1.24 to 1.65)	1.43 (1.26 to 1.63)	1.95 (1.60 to 2.39)	2.14 (1.82 to 2.51)	2.26 (1.65 to 3.09)	2.76 (2.19 to 3.47)
Bariatric Surgery	0.46 (0.36 to 0.58)	0.54 (0.45 to 0.66)	1.07 (0.88 to 1.29)	1.04 (0.86 to 1.25)	1.72 (1.41 to 2.09)	1.54 (1.27 to 1.89)	2.20 (1.72 to 2.81)	1.99 (1.54 to 2.57)
**Atherosclerosis**								
No Bariatric Surgery	1.83 (1.71 to 1.97)	1.80 (1.68 to 1.94)	3.16 (2.89 to 3.46)	3.01 (2.77 to 3.27)	4.37 (3.85 to 4.95)	4.35 (3.90 to 4.85)	5.99 (4.91 to 7.31)	6.17 (5.30 to 7.18)
Bariatric Surgery	0.84 (0.7 to 1.00)	1.27 (1.10 to 1.45)	1.56 (1.34 to 1.81)	2.11 (1.85 to 2.41)	2.54 (2.14 to 3.00)	3.05 (2.64 to 3.52)	3.92 (3.20 to 4.79)	4.33 (3.64 to 5.14)
**Arterial Embolism and Thrombosis**								
No Bariatric Surgery	0.25 (0.20 to 0.31)	0.23 (0.19 to 0.29)	0.40 (0.31 to 0.52)	0.41 (0.33 to 0.52)	0.53 (0.39 to 0.73)	0.60 (0.44 to 0.81)	0.53 (0.39 to 0.73)	0.60 (0.44 to 0.81)
Bariatric Surgery	0.11 (0.07 to 0.18)	0.14 (0.10 to 0.21)	0.25 (0.17 to 0.37)	0.25 (0.17 to 0.37)	0.35 (0.23 to 0.55)	0.37 (0.25 to 0.55)	0.35 (0.23 to 0.55)	0.37 (0.25 to 0.55)

© 2022 Elsaid MI et al. *JAMA Network Open.*

[a] Bariatric surgery status was modeled as a time-dependent covariate
[b] Angina pectoris, complications following myocardial infarction, acute coronary thrombosis, Dressler's syndrome, or chronic ischemic heart diseases
[c] Occlusion and stenosis of precerebral or cerebral arteries not resulting in ischemic stroke, cerebral atherosclerosis, acute cerebrovascular insufficiency, or cerebral ischemia
[d] Using inverse probability of treatment weights adjusted for age, health insurance type, region of residence, year of nonalcoholic fatty liver disease diagnosis, gender, smoking, asthma, obstructive sleep apnea, obesity hypoventilation syndrome, severe urinary incontinence, chronic venous insufficiency, osteoarthritis, diabetes, hypertension, dyslipidemia, chronic kidney disease, and cancer

© 2022 Elsaid MI et al. *JAMA Network Open.*

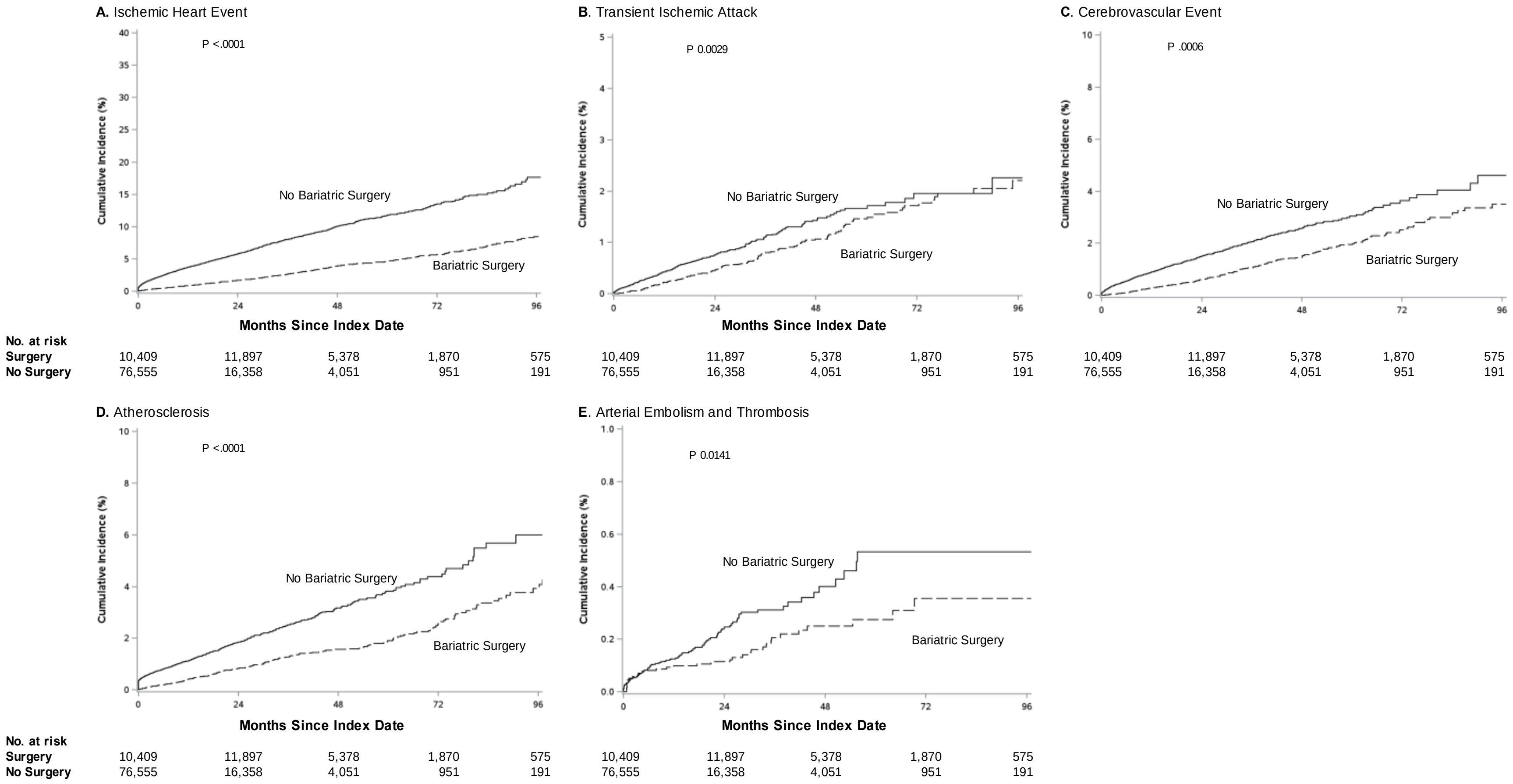

eFigure 4. Cumulative Incidence of A) Ischemic Heart Event B) Transient Ischemic Attack C) Cerebrovascular Event D) Atherosclerosis E) Arterial Embolism and Thrombosis

Bariatric surgery status was modeled as a time-dependent variable. Survival estimates were obtained using the Simon-Makuch method. P values were obtained from the Mantel and Byar test for survival comparisons of data with a time-dependent covariate.

© 2022 Elsaid MI et al. *JAMA Network Open.*

eMethods 2. Sensitivity Analyses

We conducted multiple sensitivity analyses to assess the robustness of our main findings. First, we redefined the incident diagnoses of all eight cardiovascular disease outcomes as the presence of at least two separate inpatient or outpatient claims for each outcome made ≥90 days following the first date of NAFLD diagnosis (**eTable 5**). Second, we expanded our cohort to include all NAFLD patients with BMI ≥ 30.0 Kg/M^2. We then estimated the inverse probability of treatment weights (IPTW) using inverse probability (IP) weights drawn from the expanded cohort (n=126,341). Next, we utilized the IPTW drawn for the expanded cohort to examine the association between the risk of cardiovascular disease outcomes and bariatric Surgery (**eTable 6**).

Third, we conduct a sensitivity analysis using the inverse probability of censoring weighting (IPTCW) to examine the effects of any potential selection bias due to informative censoring (6). As such, we used logistic regression models to estimate the probability of not being censored using patients' medical history and demographics. This model included all variables listed in table one with the addition of attained age at the end of follow-up, follow-up duration, surgery status, socioeconomic status, in-hospital mortality, pregnancy status, and employment status. Next, we further corrected the initially calculated IP weights of receiving surgery by multiplied them by the probability of not being censored to obtain the IPTCW (7). Subsequently, we used the IPTCW in Cox proportional hazard regression analyses to estimate the adjusted relationship between bariatric surgery and the risk of cardiovascular disease (**eTable 7**). Finally, we limited bariatric surgeries to Roux-en-Y gastric bypass and sleeve gastrectomy (**eTable 8**).

© 2022 Elsaid MI et al. *JAMA Network Open.*

eTable 5. Associations Between Bariatric Surgery and Risk of Cardiovascular Disease Outcomes in Adults With Nonalcoholic Fatty Liver Disease and Severe Obesity, 2008-2017 (n=86,964)

Outcomes	Number of Events[e]	Unadjusted	Adjusted[d]
		HR (95% CI)	HR (95% CI)
**Any Cardiovascular Event**	6733	0.45 (0.42 to 0.48)	0.48 (0.45 to 0.52)
**Primary Outcomes**	2526	0.46 (0.41 to 0.51)	0.51 (0.46 to 0.56)
Myocardial Infarction	463	0.69 (0.55 to 0.87)	0.80 (0.63 to 1.00)
Heart failure	1519	0.32 (0.27 to 0.37)	0.35 (0.30 to 0.41)
Ischemic Stroke	544	0.68 (0.56 to 0.82)	0.76 (0.62 to 0.92)
**Secondary Outcomes**	4989	0.45 (0.41 to 0.48)	0.47 (0.44 to 0.51)
Secondary Ischemic Heart Event[b]	2921	0.35 (0.32 to 0.39)	0.36 (0.33 to 0.41)
Secondary Cerebrovascular Event[c]	651	0.55 (0.45 to 0.66)	0.60 (0.49 to 0.73)
Transient Ischemic Attack	441	0.75 (0.60 to 0.93)	0.73 (0.58 to 0.91)
Atherosclerosis	867	0.52 (0.43 to 0.63)	0.62 (0.51 to 0.75)
Arterial Embolism and Thrombosis	109	0.65 (0.40 to 1.02)	0.65 (0.39 to 1.03)

[a] Bariatric surgery status was modeled as a time-dependent covariate

[b] Angina pectoris, complications following myocardial infarction, acute coronary thrombosis, Dressler's syndrome, or chronic ischemic heart diseases

[c] Occlusion and stenosis of precerebral or cerebral arteries not resulting in ischemic stroke, cerebral atherosclerosis, acute cerebrovascular insufficiency, or cerebral ischemia

[d] Using inverse probability of treatment weights adjusted for age, health insurance type, region of residence, year of nonalcoholic fatty liver disease diagnosis, gender, smoking, asthma, obstructive sleep apnea, obesity hypoventilation syndrome, severe urinary incontinence, chronic venous insufficiency, osteoarthritis, diabetes, hypertension, dyslipidemia, chronic kidney disease, and cancer

[e] At least two separate inpatient or outpatient claims made ≥90 days following the first date of NAFLD diagnosis

HR = hazard ratio; CI = confidence interval

© 2022 Elsaid MI et al. *JAMA Network Open.*

eTable 6. Associations Between Bariatric Surgery and Risk of Cardiovascular Disease Outcomes in Overweight Adults With Nonalcoholic Fatty Liver Disease, 2008-2017 (n=123,341)

**Outcomes**	**Number of Events**	**Unadjusted**	**Adjusted[d]**
		**HR (95% CI)**	**HR (95% CI)**
**Any Cardiovascular Event**	11352	0.52 (0.49 to 0.55)	0.56 (0.53 to 0.59)
**Primary Outcomes**	3560	0.61 (0.56 to 0.67)	0.54 (0.49 to 0.59)
Myocardial Infarction	618	0.68 (0.55 to 0.85)	0.81 (0.65 to 1.01)
Heart failure	2142	0.42 (0.37 to 0.48)	0.49 (0.42 to 0.55)
Ischemic Stroke	800	0.75 (0.63 to 0.89)	0.82 (0.68 to 0.97)
**Secondary Outcomes**	8734	0.50 (0.47 to 0.54)	0.54 (0.50 to 0.57)
Secondary Ischemic Heart Event[b]	4853	0.40 (0.36 to 0.44)	0.40 (0.36 to 0.44)
Secondary Cerebrovascular Event[c]	1294	0.59 (0.51 to 0.69)	0.62 (0.52 to 0.72)
Transient Ischemic Attack	663	0.81 (0.67 to 0.97)	0.76 (0.61 to 0.93)
Atherosclerosis	1741	0.61 (0.52 to 0.70)	0.80 (0.70 to 0.92)
Arterial Embolism and Thrombosis	183	0.71 (0.47 to 1.03)	0.67 (0.43 to 1.00)

[a] Bariatric surgery status was modeled as a time-dependent covariate for adults with NAFLD and BMI ≥ 35.0 kg/m^2

[b] Angina pectoris, complications following myocardial infarction, acute coronary thrombosis, Dressler's syndrome, or chronic ischemic heart diseases

[c] Occlusion and stenosis of precerebral or cerebral arteries not resulting in ischemic stroke, cerebral atherosclerosis, acute cerebrovascular insufficiency, or cerebral ischemia

[d] Using inverse probability of treatment weights adjusted for age, health insurance type, region of residence, year of nonalcoholic fatty liver disease diagnosis, gender, smoking, asthma, obstructive sleep apnea, obesity hypoventilation syndrome, severe urinary incontinence, chronic venous insufficiency, osteoarthritis, diabetes, hypertension, dyslipidemia, chronic kidney disease, and cancer

HR = hazard ratio; CI = confidence interval

© 2022 Elsaid MI et al. *JAMA Network Open.*

eTable 7. Inverse Probability of Censoring Weight Adjusted Associations Between Bariatric Surgery and Risk of Cardiovascular Outcomes in Overweight Adults With Nonalcoholic Fatty Liver Disease, 2008-2017 (n=86,964)

**Outcomes**	**Number of Events**	**Unadjusted**	**Adjusted**[d]
		**HR (95% CI)**	**HR (95% CI)**
**Any Cardiovascular Event**	8783	0.64 (0.60 to 0.67)	0.71 (0.67 to 0.76)
**Primary Outcomes**[b]	2950	0.71 (0.64 to 0.78)	0.83 (0.75 to 0.92)
**Secondary Outcomes**[c]	6615	0.61 (0.57 to 0.65)	0.69 (0.64 to 0.73)

[a] Bariatric surgery status was modeled as a time-dependent covariate for adults with NAFLD and BMI ≥ 35.0 kg/m^2

[b] Angina pectoris, complications following myocardial infarction, acute coronary thrombosis, Dressler's syndrome, or chronic ischemic heart diseases

[c] Occlusion and stenosis of precerebral or cerebral arteries not resulting in ischemic stroke, cerebral atherosclerosis, acute cerebrovascular insufficiency, or cerebral ischemia

[d] Using inverse probability of censoring weights adjusted for attained age, health insurance type, region of residence, year of nonalcoholic fatty liver disease diagnosis, gender, smoking, asthma, obstructive sleep apnea, obesity hypoventilation syndrome, severe urinary incontinence, chronic venous insufficiency, osteoarthritis, diabetes, hypertension, dyslipidemia, chronic kidney disease, cancer, follow-up duration, surgery status, socioeconomic status, in hospital mortality, pregnancy status, and employment status.

HR = hazard ratio; CI = confidence interval

© 2022 Elsaid MI et al. *JAMA Network Open.*

**eTable 8.** Associations Between Roux-en-Y Gastric Bypass and Sleeve Gastrectomy Bariatric Surgeries and Risk of Cardiovascular Disease Outcomes in Adults With Nonalcoholic Fatty Liver Disease and Severe Obesity, 2008-2017 (n=74,831)			
**Outcomes**	**Number of Events**	**Unadjusted**	**Adjusted[d]**
		**HR (95% CI)**	**HR (95% CI)**
**Any Cardiovascular Event**	7510	0.48 (0.45 to 0.52)	0.51 (0.47 to 0.55)
**Primary Outcomes**	2560	0.48 (0.43 to 0.54)	0.51 (0.46 to 0.58)
Myocardial Infarction	400	0.71 (0.54 to 0.92)	0.75 (0.57 to 0.97)
Heart failure	1624	0.35 (0.29 to 0.410	0.38 (0.32 to 0.44)
Ischemic Stroke	536	0.72 (0.58 to 0.41)	0.77 (0.32 to 0.95)
**Secondary Outcomes**	5623	0.47 (0.44 to 0.51)	0.50 (0.46 to 0.54)
Secondary Ischemic Heart Event[b]	3149	0.37 (0.33 to 0.42)	0.38 (0.34 to 0.43)
Secondary Cerebrovascular Event[c]	835	0.59 (0.49 to 0.52)	0.64 (0.53 to 0.76)
Transient Ischemic Attack	443	0.74 (0.58 to 0.92)	0.73 (0.57 to 0.92)
Atherosclerosis	1070	0.57 (0.48 to 0.68)	0.66 (0.55 to 0.78)
Arterial Embolism and Thrombosis	126	0.61 (0.37 to 0.97)	0.63 (0.38 to 1.00)

[a] Surgery status was modeled as a time-dependent covariate

[b] Angina pectoris, complications following myocardial infarction, acute coronary thrombosis, Dressler's syndrome, or chronic ischemic heart diseases

[c] Occlusion and stenosis of precerebral or cerebral arteries not resulting in ischemic stroke, cerebral atherosclerosis, acute cerebrovascular insufficiency, or cerebral ischemia

[d] Using inverse probability of treatment weights adjusted for age, health insurance type, region of residence, year of nonalcoholic fatty liver disease diagnosis, gender, smoking, asthma, obstructive sleep apnea, obesity hypoventilation syndrome, severe urinary incontinence, chronic venous insufficiency, osteoarthritis, diabetes, hypertension, dyslipidemia, chronic kidney disease, and cancer

HR = hazard ratio; CI = confidence interval

© 2022 Elsaid MI et al. *JAMA Network Open.*

eMethods 3. The E-value and Bias Factors Sensitivity Analyses for Unmeasured Confounding

While we controlled for known confounders in our main and sensitivity analyses, the nature of the claims data used in the study might leave room for unmeasured confounding. In response, we examined the potential impacts of any unmeasured confounders on our main findings by computing the unmeasured confounders bias factors and the E-values (8, 9). The bias factor is the maximum relative amount by which an unmeasured confounder could reduce an observed risk or hazard ratio (8). To estimate the bias factor, the maximum risk ratio for the unmeasured confounders-outcome association (RR_{UD}) and the risk ratio for the maximum exposure-confounders relationship (RR_{EU}) need to be specified (**Equation S1**).

To obtain conservative reference values for the confounders-outcome association, we fitted bivariate Cox proportional hazard models for the associations between smoking status, diabetes, hypertension, and dyslipidemia and each of the study outcomes (i.e., any, primary, and secondary cardiovascular outcomes, as well as, for each of the eight endpoints). We also fitted bivariate logistic regression models with surgery status as the outcome and smoking status, diabetes, hypertension, and dyslipidemia each as exposures to ascertain the reference values for the exposure-confounder relationship **(eTable 9)**. Next, we used the highest hazard and odds ratio point estimates obtained from the fitted models as inputs in equation S1 to quantify the bias factor associated with an unmeasured confounder that has the same strength of association as the highest crude impacts of known cardiovascular disease risk factors and predictors of undergoing bariatric surgery. We then divided the observed point estimates and the limits of the confidence intervals by the estimates bias factor to obtain the maximum values by which the unmeasured confounder could move the point estimates and confidence intervals towards the null – corrected estimates **(eTable 9)**.

$$B = \frac{RR_{UD}RR_{EU}}{(RR_{UD} + RR_{EU} - 1)} \qquad (Equation\ S1)$$

The E-value is a validated measure of the observed associations' robustness to potential unmeasured confounders (10). The E-value is the minimum value of the association on the risk ratio scale that an unmeasured confounder would need to have with both the outcome and exposure conditional on the measured covariates to fully explain away a specific exposure-outcome association (**Equation S2-1 & S2-2**) (8). The higher the estimated E-value for a particular association, the stronger the unmeasured confounder needs to be to explain away the observed effects (point estimate becomes the null). As such, unmeasured confounders with weaker association magnitude than the E-value cannot explain the observed association.

$$E - Vlaue = RR + \sqrt{RR(RR-1)}$$

$$for\ RR < 1\ \ let\ RR^* = \frac{1}{RR}$$

$$E - Vlaue\ (point\ estimate) = RR^* + \sqrt{RR^*(RR^*-1)} \qquad (Equation\ S2-1)$$

$$if\ Upper\ confidence\ Limit\ (UL) \geq 1, then\ E - value = 1$$

© 2022 Elsaid MI et al. *JAMA Network Open.*

$$if\ UL < 1, \quad let\ UL^* = \frac{1}{UL}$$

$$E - value\ (UL) = UL^* \times \sqrt{UL^*(UL^* - 1)} \qquad (Equation\ S2 - 2)$$

eTable 9 shows the corrected estimates relative to observed results for all the study outcomes. After assuming an unmeasured confounder with the same magnitude of association as the strongest known risk factor for cardiovascular disease, the corrected estimates show that bariatric surgery remained significantly associated with a lower risk of any, primary, and secondary cardiovascular outcomes. Furthermore, surgery remained significantly associated with lower risks for heart failure, secondary ischemic heart events, secondary cerebrovascular events, and atherosclerosis.

In our study, the observed associations between bariatric surgery and the risks of any, primary, and secondary cardiovascular outcomes were hazard ratios (HRs) 0.51 (95% confidence interval [CI] 0.48 to 0.54), 0.53 (95% CI: 0.48 to 0.59), and 0.50 (95% CI: 0.46 to 0.53) respectively. Based on the estimated E-value for the risk of cardiovascular disease, the HR of 0.51 could be fully explained (i.e., HR becomes 1) by an unmeasured confounder that has an HR of 2.56 associated with both bariatric surgery and cardiovascular disease, in addition to the confounders we adjusted for in the analysis (**eTable 10)**. Moreover, an unmeasured confounder needs at least an HR 2.43-fold association with bariatric surgery and cardiovascular disease for the observed 95% CI to contain the null value of one. To put these E-values in perspective, the crude association of any cardiovascular disease and primary cardiovascular outcomes in our sample were HR 1.36 (95% CI: 1.26 to 1.47) and HR 1.65 (1.45 to 1.85) for those with vs. without diabetes (**eTable 10)**.

© 2022 Elsaid MI et al. *JAMA Network Open.*

eTable 9. Corrected Estimates and Confidence Intervals for Unmeasured Confounding for the Effect of Bariatric Surgery[a] on the Risk of Cardiovascular Outcomes in Severely Obese Adults With Nonalcoholic Fatty Liver Disease, 2008-2017 (n=86,964)

**Outcome Category**	**Smoking History Yes vs. No HR (95% CI)**	**Diabetes Yes vs. No HR (95% CI)**	**Hypertension Yes vs. No HR (95% CI)**	**Dyslipidemia No vs. Yes HR (95% CI)**	**Study Reported Estimates[d] HR (95% CI)**	**Study Corrected Estimates HR (95% CI)**
**All Cardiovascular Outcome**	**1.36 (1.26 to 1.47)**	1.35 (1.29 to 1.41)	1.28 (1.22 to 1.35)	0.97 (0.93 to 1.01)	0.51 (0.48 to 0.54)	0.56 (0.53 to 0.60)
**Primary Outcomes**	**1.65 (1.45 to 1.85)**	1.51 (1.40 to 1.63)	1.41 (1.30 to 1.54)	1.17 (1.08 to 1.26)	0.53 (0.48 to 0.59)	0.61 (0.55 to 0.68)
Myocardial Infarction	**1.97 (1.46 to 2.60)**	1.46 (1.20 to 1.77)	1.58 (1.27 to 1.96)	1.22 (1.01 to 1.49)	0.80 (0.63 to 1.00)	0.95 (0.75 to 1.19)
Heart Failure	**1.63 (1.39 to 1.89)**	1.49 (1.35 to 1.64)	1.39 (1.25 to 1.55)	1.19 (1.08 to 1.31)	0.39 (0.34 to 0.45)	0.45 (0.39 to 0.52)
Ischemic Stroke	1.48 (1.11 to 1.92)	**1.62 (1.37 to 1.91)**	1.38 (1.15 to 1.66)	1.10 (0.93 to 1.30)	0.79 (0.66 to 0.94)	0.90 (0.76 to 1.08)
**Secondary Outcomes**	1.31 (1.20 to 1.43)	**1.33 (1.26 to 1.40)**	1.25 (1.18 to 1.32)	1.11 (1.06 to 1.17)	0.50 (0.46 to 0.53)	0.54 (0.50 to 0.58)
Secondary Ischemic Heart Event[b]	1.18 (1.04 to 1.33)	**1.38 (1.29 to 1.47)**	1.25 (1.16 to 1.34)	1.13 (1.05 to 1.21)	0.38 (0.34 to 0.42)	0.42 (0.37 to 0.46)
Secondary Cerebrovascular Event[c]	1.23 (0.97 to 1.55)	**1.30 (1.14 to 1.49)**	1.20 (1.04 to 1.39)	1.13 (0.99 to 1.29)	0.60 (0.51 to 0.70)	0.65 (0.55 to 0.76)
Transient Ischemic Attack	**1.79 (1.34 to 2.35)**	1.25 (1.04 to 1.50)	1.28 (1.06 to 1.56)	1.02 (0.85 to 1.22)	0.72 (0.59 to 0.89)	0.84 (0.69 to 1.04)
Atherosclerosis	**1.52 (1.25 to 1.83)**	1.27 (1.13 to 1.43)	1.34 (1.18 to 1.53)	1.16 (1.03 to 1.31)	0.70 (0.61 to 0.81)	0.79 (0.69 to 0.91)
Arterial Embolism and Thrombosis	**1.75 (1.01 to 2.83)**	1.10 (0.76 to 1.57)	1.08 (0.76 to 1.53)	1.43 (1.01 to 2.05)	0.61 (0.40 to 0.91)	0.71 (0.47 to 1.06)

[a] Bariatric surgery status was modeled as a time-dependent covariate for adults with NAFLD and BMI ≥ 40.0 kg/m^2

[b] Angina pectoris, complications following myocardial infarction, acute coronary thrombosis, Dressler's syndrome, or chronic ischemic heart diseases

[c] Occlusion and stenosis of precerebral or cerebral arteries not resulting in ischemic stroke, cerebral atherosclerosis, acute cerebrovascular insufficiency, or cerebral ischemia

[d] Using inverse probability of treatment weights adjusted for age, health insurance type, region of residence, year of nonalcoholic fatty liver disease diagnosis, gender, smoking, asthma, obstructive sleep apnea, obesity hypoventilation syndrome, severe urinary incontinence, chronic venous insufficiency, osteoarthritis, diabetes, hypertension, dyslipidemia, chronic kidney disease, and cancer.

Bolded= point estimate used as inputs for the R_{UD}, R_{EU} = 1.49 (highest point estimate for the confounder-exposure association).

HR = hazard ratio; CI = confidence interval

© 2022 Elsaid MI et al. *JAMA Network Open.*

eTable 10. Point Estimates Upper-Level Confidence Intervals and E-Values for the Effect of Bariatric Surgery[a] on the Risk of Cardiovascular Outcomes in Severely Obese Adults With Nonalcoholic Fatty Liver Disease, 2008-2017 (n=86,964)

**Outcome Category**	**Smoking History Yes vs. No HR (95% CI)**	**Diabetes Yes vs. No HR (95% CI)**	**Hypertension Yes vs. No HR (95% CI)**	**Dyslipidemia No vs. Yes HR (95% CI)**	**E-value**	
					**HR Point Estimate**	**HR 95% CI Upper limit**
**Any Cardiovascular Event**	1.36 (1.26 to 1.47)	1.35 (1.29 to 1.41)	1.28 (1.22 to 1.35)	0.97 (0.93 to 1.01)	2.56	2.43
**Primary Outcomes**	1.65 (1.45 to 1.85)	1.51 (1.40 to 1.63)	1.41 (1.30 to 1.54)	1.17 (1.08 to 1.26)	2.47	2.24
Myocardial Infarction	1.97 (1.46 to 2.60)	1.46 (1.20 to 1.77)	1.58 (1.27 to 1.96)	1.22 (1.01 to 1.49)	1.62	1.06
Heart failure	1.63 (1.39 to 1.89)	1.49 (1.35 to 1.64)	1.39 (1.25 to 1.55)	1.19 (1.08 to 1.31)	3.22	2.86
Ischemic Stroke	1.48 (1.11 to 1.92)	1.62 (1.37 to 1.91)	1.38 (1.15 to 1.66)	1.10 (0.93 to 1.30)	1.63	1.26
**Secondary Outcomes**	1.31 (1.20 to 1.43)	1.33 (1.26 to 1.40)	1.25 (1.18 to 1.32)	1.11 (1.06 to 1.17)	2.16	2.47
Secondary Ischemic Heart Event[b]	1.18 (1.04 to 1.33)	1.38 (1.29 to 1.47)	1.25 (1.16 to 1.34)	1.13 (1.05 to 1.21)	4.70	4.19
Secondary Cerebrovascular Event[c]	1.23 (0.97 to 1.55)	1.30 (1.14 to 1.49)	1.20 (1.04 to 1.39)	1.13 (0.99 to 1.29)	2.72	2.21
Transient Ischemic Attack	1.79 (1.34 to 2.35)	1.25 (1.04 to 1.50)	1.28 (1.06 to 1.56)	1.02 (0.85 to 1.22)	2.12	1.50
Atherosclerosis	1.52 (1.25 to 1.83)	1.27 (1.13 to 1.43)	1.34 (1.18 to 1.53)	1.16 (1.03 to 1.31)	2.21	1.77
Arterial Embolism and Thrombosis	1.75 (1.01 to 2.83)	1.10 (0.76 to 1.57)	1.08 (0.76 to 1.53)	1.43 (1.01 to 2.05)	2.66	1.43

[a] Bariatric surgery status was modeled as a time-dependent covariate for adults with NAFLD and BMI ≥ 40.0 kg/m^2

[b] Angina pectoris, complications following myocardial infarction, acute coronary thrombosis, Dressler's syndrome, or chronic ischemic heart diseases

[c] Occlusion and stenosis of precerebral or cerebral arteries not resulting in ischemic stroke, cerebral atherosclerosis, acute cerebrovascular insufficiency, or cerebral ischemia

HR = hazard ratio; CI = confidence interval

© 2022 Elsaid MI et al. *JAMA Network Open.*

eReferences

1. George MD, Baker JF, Winthrop K, Alemao E, Chen L, Connolly S, et al. Risk of biologics and glucocorticoids in patients with rheumatoid arthritis undergoing arthroplasty: a cohort study. Annals of internal medicine. 2019;170(12):825-36.
2. Park H, Wang W, Henry L, Nelson DR**.** Impact of All-Oral Direct-Acting Antivirals on Clinical and Economic Outcomes in Patients With Chronic Hepatitis C in the United States. Hepatology. 2019;69(3):1032-45.
3. Wernli KJ, Brenner AT, Rutter CM, Inadomi JM**.** Risks Associated With Anesthesia Services During Colonoscopy. Gastroenterology. 2016;150(4):888-e18.
4. Mechanick JI, Youdim A, Jones B, Garvey W, Hurley D, McMahon M**.** AACe/tos/AsMbs Guidelines. Endocrine practice. 2008;14(Suppl 1).
5. Mechanick JI, Youdim A, Jones DB, Garvey WT, Hurley DL, McMahon MM, et al. Clinical practice guidelines for the perioperative nutritional, metabolic, and nonsurgical support of the bariatric surgery patient—2013 update: cosponsored by American Association of Clinical Endocrinologists, the Obesity Society, and American Society for Metabolic & Bariatric Surgery. Surgery for Obesity and Related Diseases. 2013;9(2):159-91.
6. Cole SR, Hernán MA**.** Constructing inverse probability weights for marginal structural models. American journal of epidemiology. 2008;168(6):656-64.
7. Nahon P, Layese R, Bourcier V, Cagnot C, Marcellin P, Guyader D, et al. Incidence of hepatocellular carcinoma after direct antiviral therapy for HCV in patients with cirrhosis included in surveillance programs. Gastroenterology. 2018;155(5):1436-50. e6.
8. VanderWeele TJ, Ding P**.** Sensitivity analysis in observational research: introducing the E-value. Annals of internal medicine. 2017;167(4):268-74.
9. Mathur MB, Ding P, Riddell CA, VanderWeele TJ**.** Website and R package for computing E-values. Epidemiology (Cambridge, Mass.). 2018;29(5):e45.
10. Jirapinyo P, McCarty TR, Dolan RD, Shah R, Thompson CC**.** Effect of endoscopic bariatric and metabolic therapies on nonalcoholic fatty liver disease: a systematic review and meta-analysis. Clinical Gastroenterology and Hepatology. 2021.

© 2022 Elsaid MI et al. *JAMA Network Open.*
